# The 5'-poly(A) leader of poxvirus mRNA confers a translational advantage that can be achieved in cells with impaired cap-dependent translation

**DOI:** 10.1371/journal.ppat.1006602

**Published:** 2017-08-30

**Authors:** Pragyesh Dhungel, Shuai Cao, Zhilong Yang

**Affiliations:** Division of Biology, Kansas State University, Manhattan, Kansas, United States of America; University of Alberta, CANADA

## Abstract

The poly(A) leader at the 5’-untranslated region (5’-UTR) is an unusually striking feature of all poxvirus mRNAs transcribed after viral DNA replication (post-replicative mRNAs). These poly(A) leaders are non-templated and of heterogeneous lengths; and their function during poxvirus infection remains a long-standing question. Here, we discovered that a 5’-poly(A) leader conferred a selective translational advantage to mRNA in poxvirus-infected cells. A constitutive and uninterrupted 5’-poly(A) leader with 12 residues was optimal. Because the most frequent lengths of the 5’-poly(A) leaders are 8–12 residues, the result suggests that the poly(A) leader has been evolutionarily optimized to boost poxvirus protein production. A 5’-poly(A) leader also could increase protein production in the bacteriophage T7 promoter-based expression system of vaccinia virus, the prototypic member of poxviruses. Interestingly, although vaccinia virus post-replicative mRNAs do have 5’- methylated guanosine caps and can use cap-dependent translation, in vaccinia virus-infected cells, mRNA with a 5’-poly(A) leader could also be efficiently translated in cells with impaired cap-dependent translation. However, the translation was not mediated through an internal ribosome entry site (IRES). These results point to a fundamental mechanism poxvirus uses to efficiently translate its post-replicative mRNAs.

## Author summary

Poxviruses continue to impact public health significantly, despite the eradication of smallpox, the deadliest disease in human history. As a tool, poxviruses are being engineered to treat various infectious diseases and multiple cancers. All poxvirus mRNAs transcribed after viral DNA replication have a poly(A) leader in their 5’-untranslated regions, the function of which remains elusive and represents a major gap in our understanding of the mechanisms fundamental to controlling poxvirus gene expression. In poxvirus-infected cells, a 5’-poly(A) leader was found to confer on poxvirus mRNAs a translational advantage that could be achieved in cells with impaired cap-dependent translation, which is used for translation of most eukaryotic mRNAs. Furthermore, since viruses typically exploit existing cellular functions, it is highly likely that these results point to an unknown cellular mRNA translation mechanism. Thus, the findings should facilitate targeting of poxvirus post-replicative mRNA translation for the development of novel antiviral strategies. The poly(A) leader can also be used to increase foreign gene expression when using the bacteriophage T7 promoter-based poxvirus expression systems.

## Introduction

All viruses rely entirely on their infected host cells for protein synthesis. Not surprisingly, to boost production of viral proteins, many viral evolutionary strategies usurp the host translation machinery. In doing so they target every step of protein synthesis, from mRNA production and stability, to translation initiation, elongation, and termination [1–4]. Such mechanisms include mRNA sequence elements that enhance translation. A striking and unusual feature is found in all vaccinia virus (VACV) mRNAs transcribed after viral DNA replication; all of these mRNAs have a 5’-poly(A) leader in their 5’-untranslated regions (5’-UTRs) [5–8]. It is well established that the 5’-UTR of an mRNA plays an important role in regulating eukaryotic mRNA translation [9]; for the VACV mRNAs, however, it is unclear whether the 5’-poly(A) leader contributes to efficient translation of these VACV mRNAs. This lack of knowledge represents a major gap in understanding the fundamental gene expression mechanism of poxviruses.

Poxviruses comprise a highly dangerous class of emerging and re-emerging pathogens of humans and other vertebrates [10]. Their large double-stranded DNA genomes encode hundreds of genes that are expressed in cascade at early, intermediate, or late stages of infection [10]. The early genes initiate expression soon after viral entry, without the need for viral DNA replication; in contrast, intermediate and late genes can only be expressed after viral DNA replication. The intermediate and late genes are collectively referred to as post-replicative genes, and mainly function to form virions. In VACV, the prototypic poxvirus, 53 genes initiate transcription in the intermediate stage and 38 genes initiate transcription in the late stage [11, 12]. All the VACV post-replicative mRNAs contain a non-templated 5’-poly(A) leader that is likely formed during transcription initiation, when the viral RNA polymerase slips at the conserved promoter sequence containing three A residues [6–8, 13]. The 5’-poly(A) leaders of these mRNAs are of heterogeneous length ranging from 3 to 51 A residues, with most between 8 and 12 A residues [5]. For most VACV post-replicative mRNAs, the 5’-poly(A) leaders comprise the entire 5’-UTR because the 5’-poly(A) leader and the first A residue of the start codon AUG overlap [5]. Similar to eukaryotic cellular mRNAs, VACV early mRNAs and post-replicative VACV mRNAs are capped by methylated guanosine [14, 15]. The 5’ cap is required for launching cap-dependent translation that is the dominant translation mode in eukaryotic cells [16]. Therefore, like eukaryotic cellular mRNAs, these VACV mRNAs can be translated in a cap-dependent manner.

To examine the function of the VACV poly(A) leader, we first developed an in vitro transcribed RNA-based reporter assay. We used this assay to demonstrate that the 5’-poly(A) leader of an mRNA can confer a translational advantage in VACV-infected cells. Remarkably, the translational advantage can be achieved in cells with impaired cap-dependent translation, suggesting an adaptation mechanism poxvirus uses to replicate in unfriendly cellular environments.

## Results

### The 5’-poly(A) leader of an mRNA confers a translational advantage during VACV infection

To understand how the 5’-poly(A) leader may facilitate mRNA translation during VACV infection, we intended to develop a convenient reporter system. Though DNA plasmid-based luciferase expression is usually used to develop reporter system, technical issues posed barriers of the utility of it in VACV-infected cells as plasmids are able to replicate in VACV-infected cells [17]; such plasmid replication makes it difficult to compare uninfected and VACV-infected cells. In addition, VACV promoter-driven transcription would generate mRNAs that are heterogeneous with respect to poly(A)-leader length [5]; and, with plasmid templates, cryptic transcription would likely further complicate interpretation of the data [5, 11, 18].

**Fig 1:**
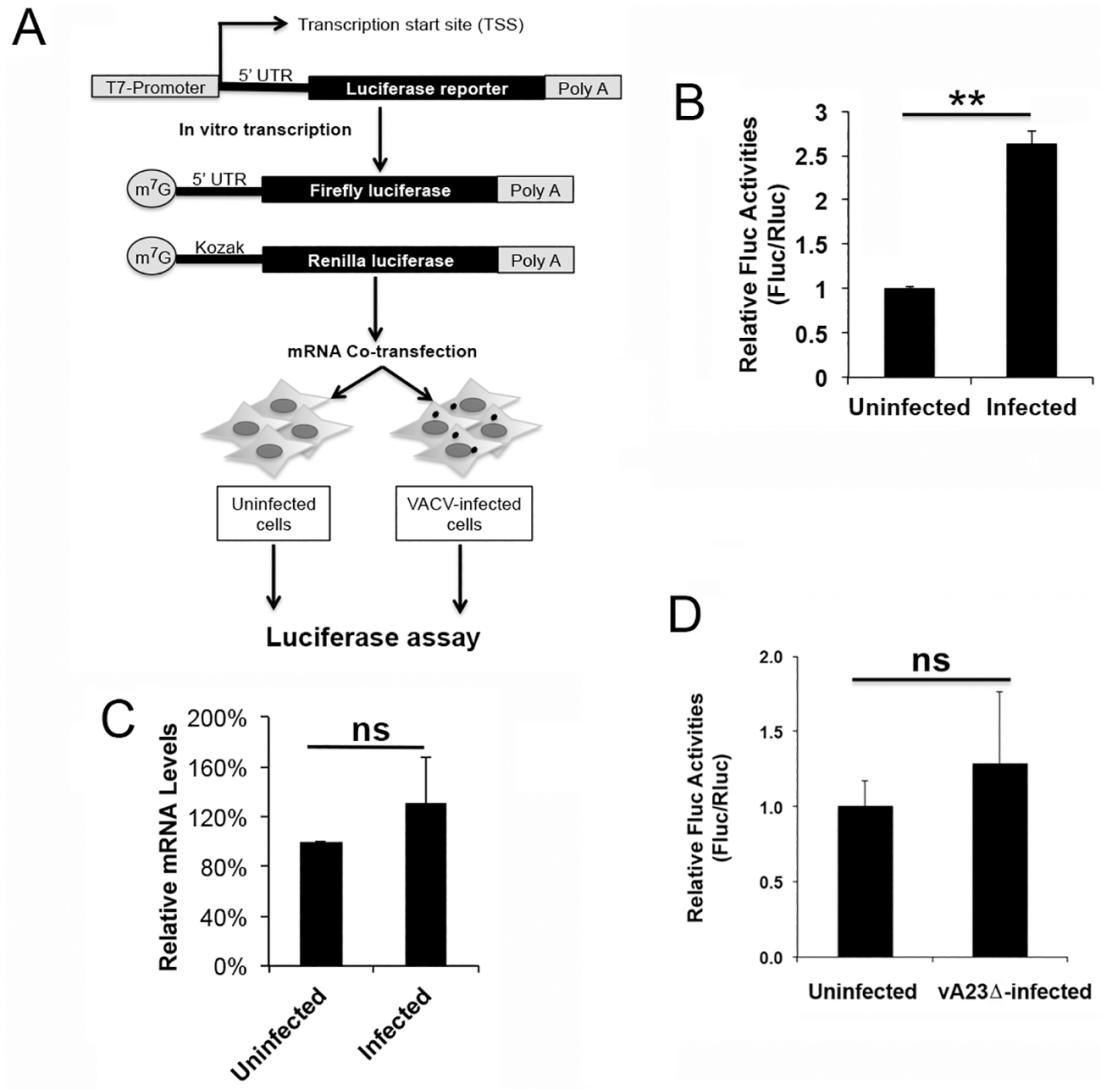
The 5’ poly(A) leader confers an mRNA translational advantage during the post-replicative stage of VACV replication. (A) Schematic of experimental approach. Messenger RNA was synthesized by a T7 promoter-based in vitro transcription system. The firefly luciferase (Fluc) reporter mRNA under a 5’-UTR to be tested was transfected into cells together with a renilla luciferase (Rluc) reporter mRNA under a 5’-UTR-containing Kozak sequence. Luciferase activities were measured at 5 h post transfection using a luminometer with a dual luciferase assay system. (B) Fluc mRNA with a 5’-poly(A) leader of 20 residues was transfected into uninfected or wild-type VACV-infected HeLa cells (12 hpi) together with an Rluc mRNA. Luciferase activities were measured at 5 h post transfection. The Rluc-normalized Fluc activity was normalized as 1 in uninfected HeLa cells. (C) The relative levels of Fluc mRNA from uninfected and VACV-infected HeLa cells were quantitated at 5 h post transfection by quantitative RT-PCR (qRT-PCR). The amount of mRNA in uninfected cells was normalized as 1. (D) Fluc mRNA with a 5’-poly(A) leader of 20 residues was transfected into uninfected, or A23-deleted recombinant VACV-infected HeLa cells (12 hpi) together with an Rluc mRNA. Luciferase activities were measured at 5 h post transfection. The Rluc-normalized Fluc activity was normalized as 1 in uninfected HeLa cells. Error bars represent standard deviation (SD) of at least three experiments. P-values were obtained using the Student’s t-test; **P value < 0.01, ns = Not Significant (i.e. P value > 0.05).

To work around these complications, we developed an RNA-based luciferase reporter assay (Fig 1A). Using PCR, we first generated a DNA fragment containing the following elements in 5’ to 3’ order: a T7 promoter, the desired 5’-UTR sequence, the firefly luciferase (Fluc) ORF, and sequence encoding a poly (A) tail. The mRNA was transcribed from a bacterial T7-phage-promoter-based, in vitro transcription system, during which an m^7^G cap or its analog was incorporated. The resulting Fluc reporter mRNA was transfected into cells, together with a renilla luciferase (Rluc) reporter mRNA as the control of transfection efficiency. The Rluc mRNA contained a 5’-UTR bearing the Kozak consensus sequence that is important in translation initiation of eukaryotic mRNA [19]. The normalized firefly luciferase activity was used as the indicator of translational potential of mRNAs with different 5’-UTRs.

**Fig 2:**
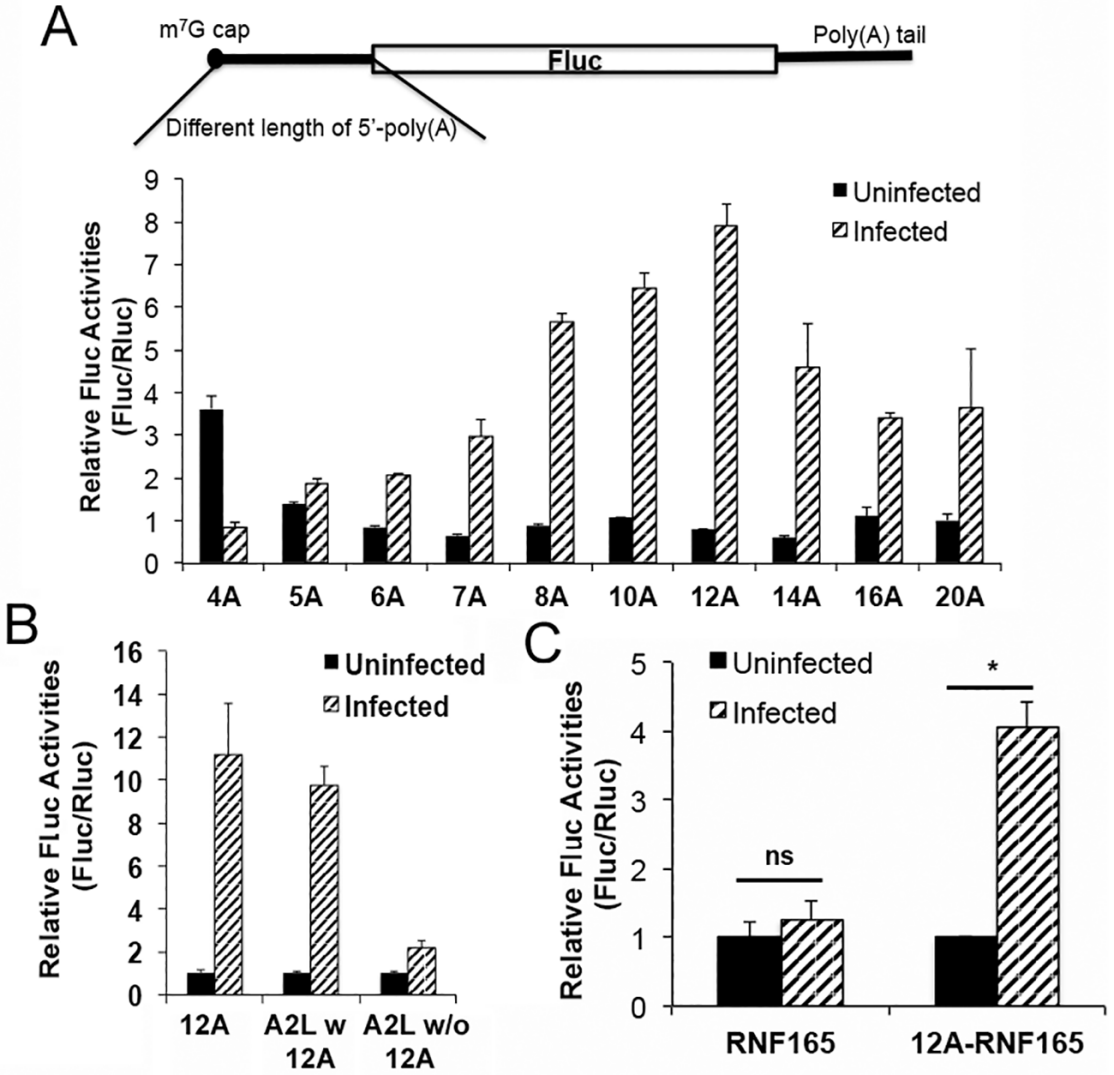
The optimal length of 5’-poly(A) leader in conferring translational advantage is 12 residues. (A) The Fluc reporter mRNAs with different 5’-poly(A)-leaders lengths were transfected into uninfected and VACV-infected HeLa cells, together with an Rluc mRNA. Luciferase activities were measured at 5 h post transfection. (B) Fluc reporter mRNA containing the A2L mRNA 5’-UTR with (w) or without (w/o) a poly(A) leader, was transfected into uninfected and VACV-infected HeLa cells, together with an Rluc mRNA. Luciferase activities were measured at 5 h post transfection. The Rluc-normalized Fluc activity was normalized as 1 in uninfected cells. (C) Fluc reporter mRNA containing RNF165 5’-UTR with (w) or without (w/o) an A-tract addition at the 5’-end was transfected into uninfected and VACV-infected HeLa cells together with an Rluc mRNA. Luciferase activities were measured at 5 h post transfection. Error bars represent standard deviation (SD) of at least three experiments. P- values were determined using the Student’s t-test; *P value < 0.05.

Using this approach, we tested the translational efficiency of an Fluc mRNA whose 5’-UTR bears a 20-residue 5’-poly(A) leader. Translational efficiency was compared in uninfected and VACV-infected HeLa cells. The transfection was carried out at approximately 12 hpi, during the VACV post-replicative stage of infection [11]. We tested the luciferase activities at 1, 2.5, 5 and 8 h post transfection. As expected, both firefly and renilla luciferase activities increased over time. At 5 h, the luciferase activities did not reach or just reached the highest value in both uninfected and infected cells (S1A and S1B Fig). In the following experiments, the luciferase activities were measured at approximately 5 h post transfection. Notably, compared to uninfected cells, VACV-infected cells showed a significant increase of the normalized Fluc activity (Fig 1B). The control 5’-UTR from the cellular RNF165 mRNA, which contains no poly(A) leader, did not confer a translational advantage in VACV-infected cells (Fig 2C). The Fluc mRNA with a poly(A) leader amounts in VACV-infected and uninfected cells at 5 h post transfection were similar, shown here by quantitative RT-PCR (Fig 1C), ruling out the possibility of difference in luciferase activities due to different amounts of transfected RNA in VACV-infected cells. Interestingly, the translational advantage was not observed during the early replication stage as evidenced by using an A23 gene-deleted VACV (A23Δ) that arrested viral replication at the DNA replication stage (Fig 1D) [20]. Together, these results showed that, in VACV-infected HeLa cells, mRNA with a 5’-poly(A) leader was more efficiently translated during the post-replicative stage of VACV replication.

### 12 residues is the optimal length of the 5’-poly(A) leader for conferring a translational advantage during VACV infection

Among VACV post-replicative mRNAs, the length of the 5’-poly(A) leader varies from 3 to 51 residues, with most leaders between 8 and 12 residues [5]. To determine the optimal length for conferring the translational advantage, we generated Fluc mRNAs with different poly(A) leader lengths, ranging from 4 to 20 residues. In VACV-infected cells, our reporter assays showed the translational advantage of the Fluc mRNAs increased as the length of the 5’-poly(A) leader increased to 12 residues (approximately 8-fold), and then decreased when leaders became longer (Fig 2A). The absolute luciferase activity was also the highest for the Fluc mRNA with a 5’-poly(A) leader of 12 residues in VACV-infected cells (S2 Fig), further indicating the optimal length for conferring a translational advantage during VACV infection was 12 residues. We also examined the translational advantage during the VACV post-replicative stage by transfecting the 12A-headed RNA at 2.5, 5, 7.5, 10 and 15 hpi. The results showed translational advantage during all the times examined (S3 Fig).

### Addition of a 5’-poly(A) tract at the 5’ end of a non-poly(A) UTR confers mRNA a translational advantage in VACV-infected cells

Although most VACV post-replicative mRNAs have a 5’-poly(A) leader immediately upstream of the start codon, some also contain a non-poly(A) intervening sequence [5], e.g., the viral A2L mRNA has a 32-nucleotide, non-poly(A) sequence between the poly(A) leader and the start codon [5]. In VACV-infected cells, we tested the A2L mRNA 5’-UTR lacking a 5’-poly(A) tract, and found that it offered only slight translational advantage (Fig 2B). Adding a 12-residue A-tract to this mRNA at the 5’ end of the 5’-UTR, however, significantly increased its expression (Fig 2B).

To test whether a poly(A) leader can enhance translation potential of non-viral 5’-UTR during VACV infection, we added 12 A residues to the 5’ end of the RNF165 5’-UTR, which on its own only slightly enhance translation in VACV-infected cells (Fig 2C). In VACV-infected cells, addition of a 5’-end, 12-nucleotide A-tract to the RNF165 5’-UTR greatly enhanced translation of a downstream Fluc reporter (Fig 2C). These results further demonstrated that a 5’-poly(A) leader conferred a translational advantage in VACV-infected cells.

### An uninterrupted 5’-poly(A) leader is essential for optimal translation in VACV-infected cells

**Fig 3:**
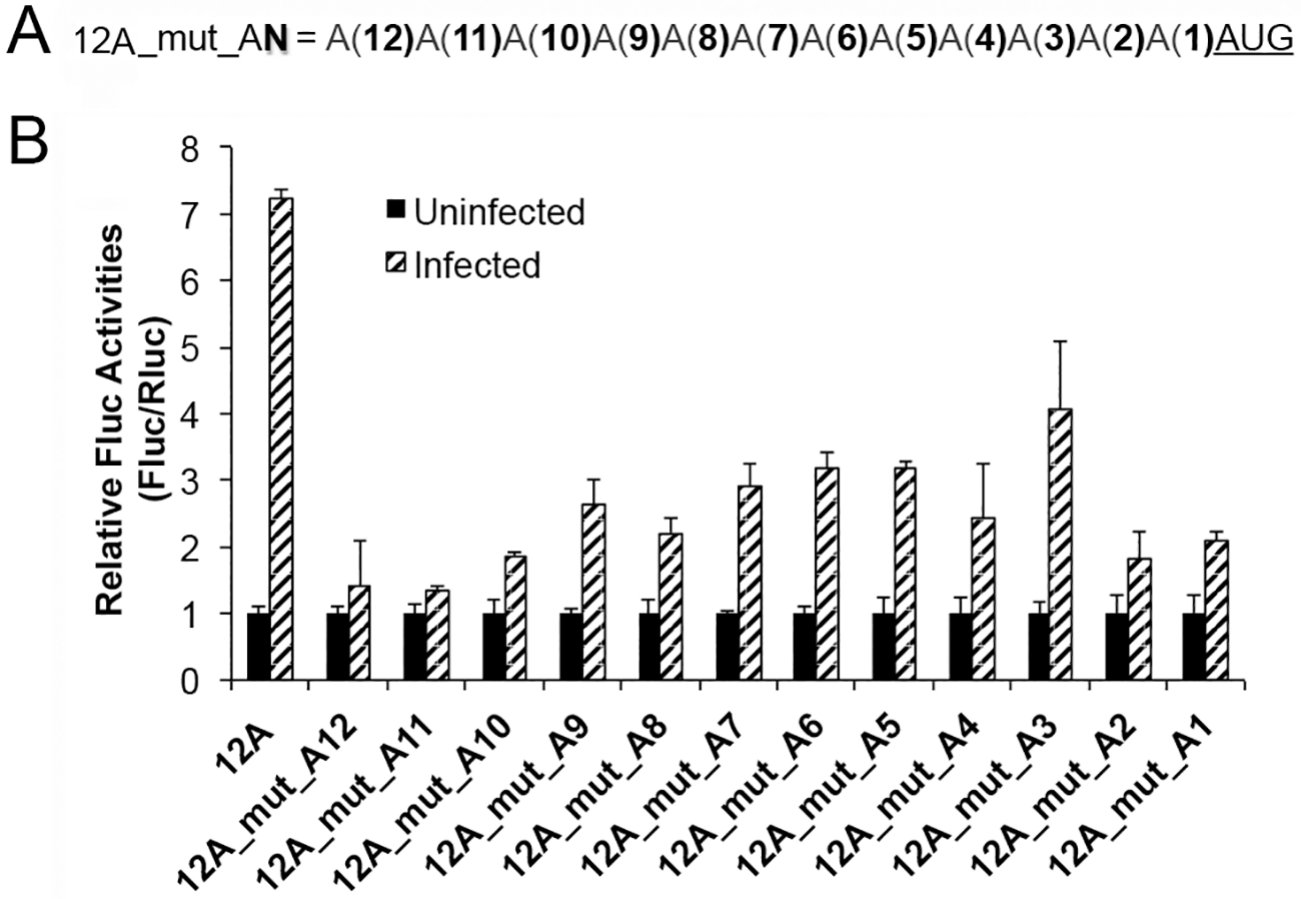
An uninterrupted 5’-poly(A) leader is essential for optimal translation in VACV-infected cells. (A) Schematic of representation showing individual 5’-poly(A) residues mutated to U. (B) Fluc reporter mRNA containing each of the mutated 5’-poly(A) leaders were transfected into uninfected and VACV-infected HeLa cells, together with an Rluc mRNA. Luciferase activities were measured at 5 h post transfection. The Rluc normalized Fluc activity was normalized as 1 in uninfected HeLa cells. Error bars represent standard deviation (SD) of at least three experiments.

We next investigated which A, amid the 12 residues, was vital for the poly(A) leader-mediated translational advantage. We generated Fluc mRNAs, with A to U point mutations at each of the 12 positions. The mutation was represented as 12A_mut_A**N**, where mutation A to U was at the position ‘N’ upstream of start codon AUG (Fig 3A). Any A to U change in the poly(A) leader affected the translational advantage (Fig 3B). We also mutated the A to G in several positions and these mutations all impaired the translational advantage (S4 Fig). This result indicated that an uninterrupted 5’-poly(A) leader is critical for the 5’-poly(A) leader-mediated translational advantage.

### A 5’-poly(A) leader confers a translational advantage in multiple cell types and in myxoma virus infection

**Fig 4:**
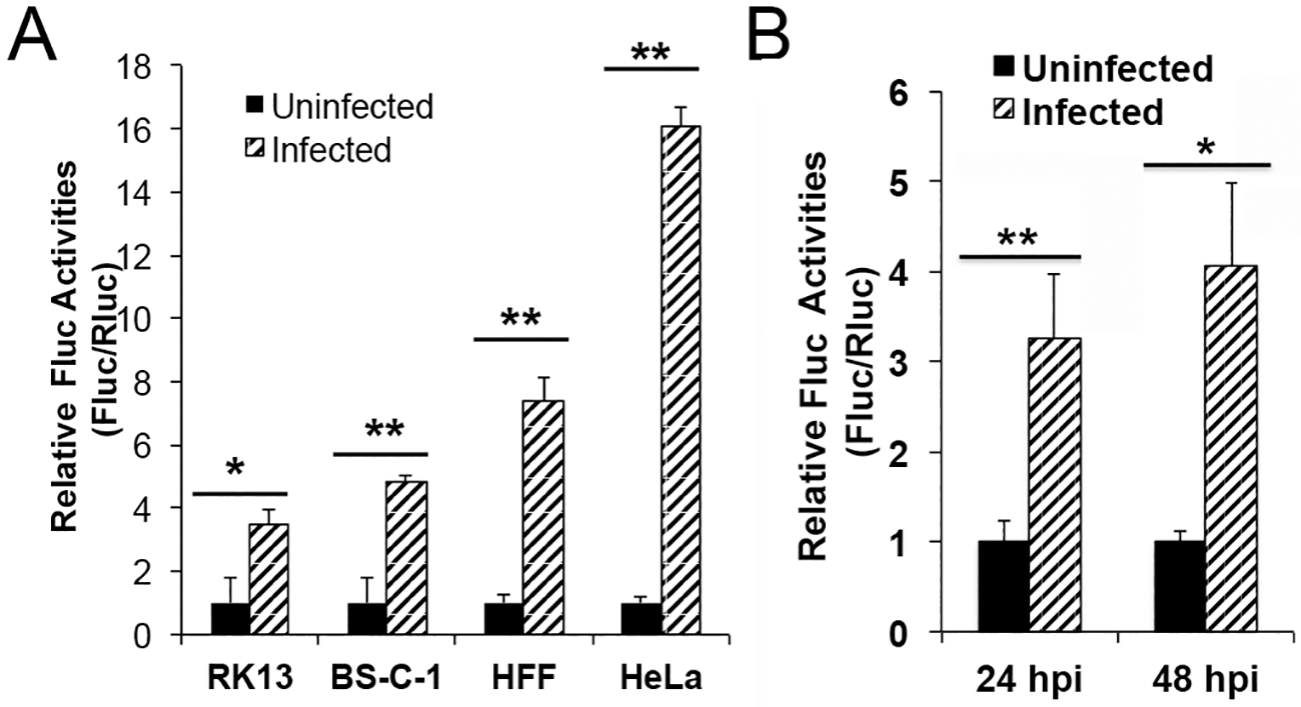
A 5’-poly(A) leader confers a translational advantage in multiple cell types and in myxoma virus infection. (A) An Fluc reporter mRNA with a 12-residue 5’-poly(A) leader was transfected into uninfected or VACV-infected HeLa, HFF, BS-C-1 or RK13 cells, together with an Rluc mRNA, at 24 and 48 hpi. Luciferase activities were measured at 5 h post transfection. The Rluc-normalized Fluc activity was normalized as 1 in uninfected cells. (B) An Fluc reporter mRNA with a 12-residue 5’-poly(A) leader was transfected into uninfected or myxoma virus-infected RK13 cells, together with an Rluc mRNA. Luciferase activities were measured at 5 h post transfection. Error bars represent standard deviation (SD) of at least three experiments. P-values were determined using the Student’s t-test; **P value < 0.01, *P value < 0.05.

We examined the effect of the 5’-poly(A) leader on translation in different cell lines infected with VACV. During VACV infection, the translational upregulation of mRNA with a poly(A) leader was also observed in human foreskin fibroblasts (HFFs), rabbit RK13, and monkey BS-C-1, though the enhancement levels varied (Fig 4A). We also tested the effect of the 5’-poly(A) leader on translation in RK-13 cells infected with Myxoma virus, a leporipoxvirus, and it similarly enhanced translational efficiency (Fig 4B).

### Messenger RNA with a 5’-poly(A) leader is more efficiently translated than mRNA without a 5’-poly(A) leader in VACV-infected cells

**Fig 5:**
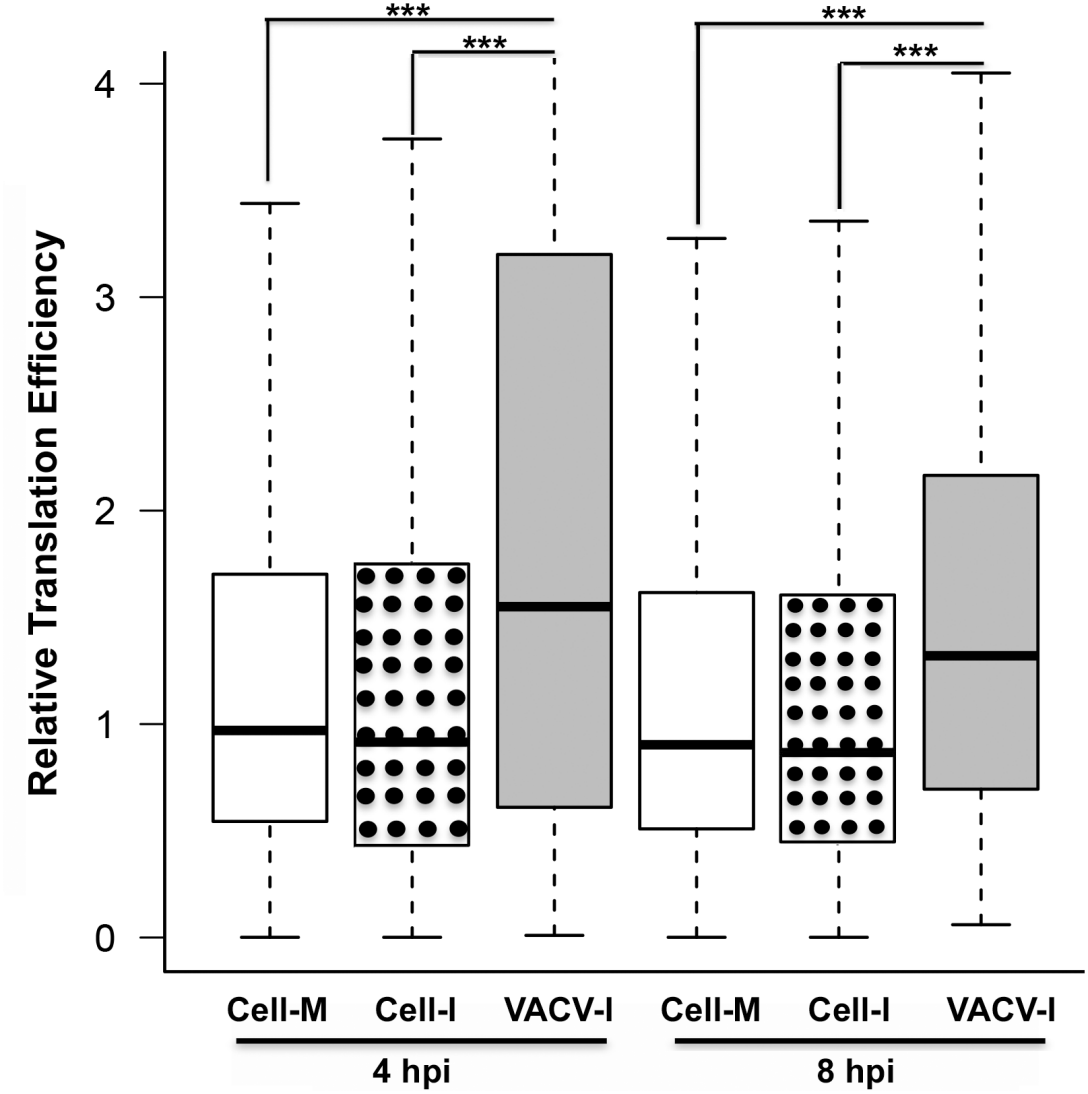
VACV post-replicative mRNAs are more efficiently translated than cellular mRNAs. Boxwhisker plots of relative translation efficiency of VACV post-replicative mRNAs and host mRNAs at 4 and 8 hpi. Relative translation efficiency of mRNAs from uninfected HeLa cells is also shown. The bottom and top of the box indicate the first and third quartiles, respectively, with the line in the middle representing the median. Cell-M, cellular mRNAs from mock-infected cells. Cell-I, cellular mRNAs from VACV-infected cells. VACV-I, VACV mRNAs. The highest relative translation efficiency of VACV-I was off scale. ***indicates a P value < 0.001.

We investigated whether, in VACV-infected cells, mRNAs containing a 5’-poly(A) leader have a translational advantage over those without a 5’-poly(A) leader. In previous study, we monitored the levels of active mRNA translation in VACV-infected HeLa cells by simultaneous mRNA sequencing (RNA-Seq) and ribosome profiling [21, 22]. Here, we calculated the genome-wide, relative translation efficiency (TE) of all VACV post-replicative mRNAs by the ratio of ribosome-protected mRNA to total mRNA, mapped to each transcript during the post-replicative stage at 4 and 8 hpi. VACV replication had proceeded to the post-replicative stage at 4 hpi under the experimental condition, when most viral mRNAs have 5’-poly(A) leaders [21]. Although this calculation likely underestimated VACV mRNA translation efficiency, due to extensive read-through of VACV post-replicative mRNAs [5, 12], the VACV mRNAs were translated significantly more efficiently than cellular mRNAs (both in mock and VACV-infected cells), in a genome-wide manner (Fig 5). In fact, although there was an overall decrease of mRNA translation efficiency at 8 hpi compared to 4 hpi in VACV-infected cells, the translation efficiency of viral post-replicative mRNAs was still significantly higher than that of cellular mRNAs in mock-infected cells. These results indicated that, during a VACV infection, the post-replicative mRNAs with 5’-poly(A) leaders were more efficiently loaded with ribosomes, indicating higher translation efficiency of viral post-replicative mRNAs than that of host mRNAs (without 5’-poly(A) leaders).

**Fig 6:**
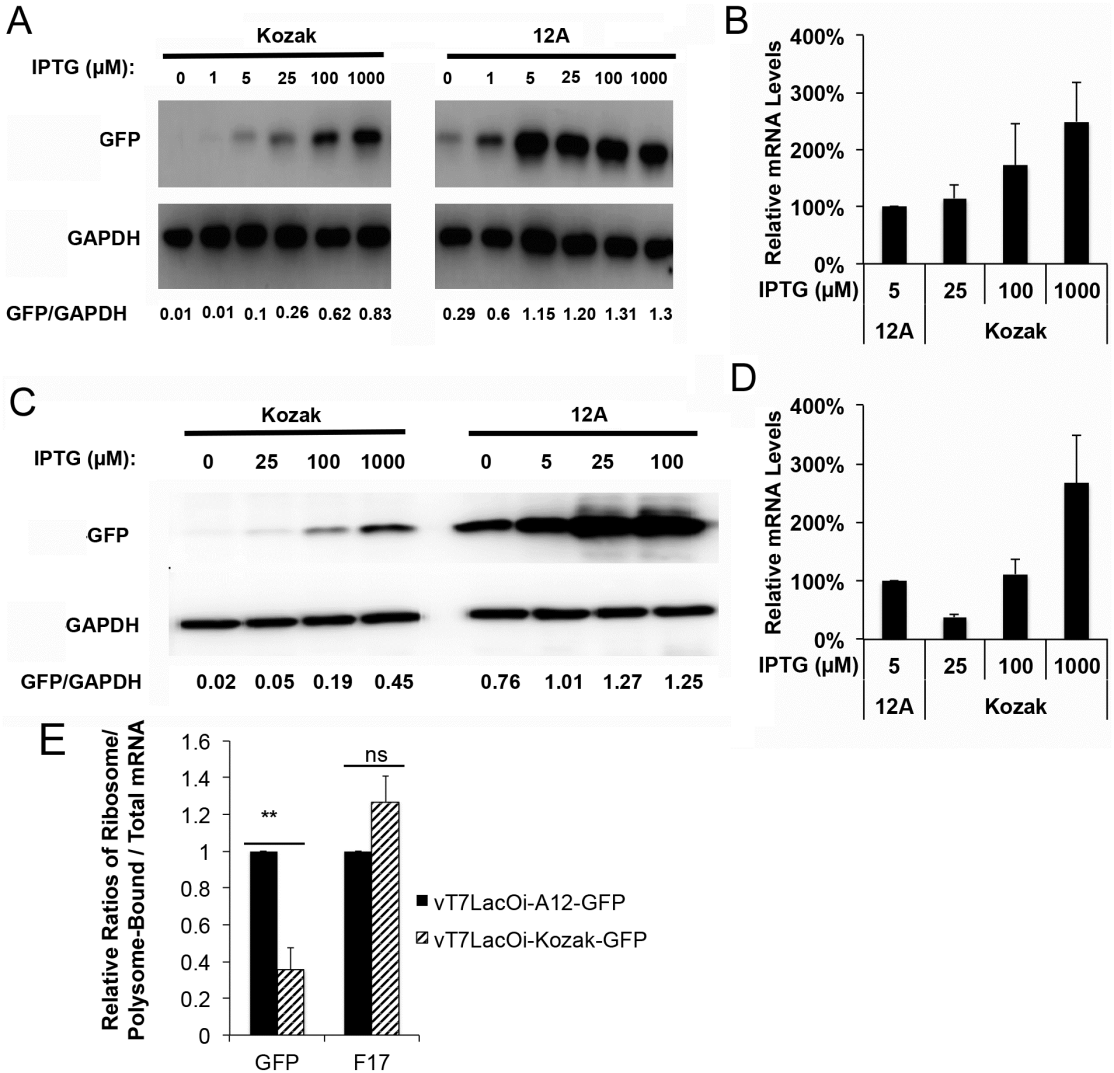
Messenger RNA with a 5’-poly(A) leader is more efficiently translated than mRNA without a 5’-poly(A) leader in VACV-infected cells. (A) HeLa cells were transfected with plasmids expressing eGFP from a T7 promoter and containing an A-tract or a Kozak sequence between the ATG and the T7 promoter. Cells were infected with vT7LacOi 24 h post transfection and different concentrations of IPTG were added to the media as indicated. At 24 hpi, Western blotting tracked eGFP and GAPDH (loading control) expression levels; and eGFP intensity was normalized to GAPDH, shown below. (B) Levels of eGFP mRNA levels for (A) were measured by qRT-PCR, and normalized to 18S rRNA. (C) HeLa cells were infected with recombinant VACV vT7LacOi_Kozak-GFP or vT7LacOi_A12-GFP. Indicated concentrations of IPTG were added to the culture media. At 24 hpi Western blotting tracked eGFP and GAPDH (normalization and loading control) expression, shown below. (D) Levels of eGFP mRNA expression of (C) were measured by qRT-PCR, and normalized to 18S rRNA. (E) HeLa cells were infected with vT7LacOi_A12-GFP or vT7LacOi_Kozak-GFP. Total and ribosome/polysome-bound RNAs were isolated at 15 hpi. The GFP and F17 mRNAs were measured by qRT-PCR and normalized to 18S rRNA. The ratios of ribosome/polysome-bound mRNA to total mRNA were calculated for GFP and F17, respectively. The ratios from vT7LacOi_A12-GFP-infected cells were normalized to one. **indicates a P value < 0.01. ns = Not Significant (i.e. P value > 0.05).

To examine further the role of the 5’-poly(A) leader, we carried out two experiments. In the first we constructed two plasmids in which an eGFP gene was under the control of the T7 promoter. An A-tract or a sequence containing a Kozak element was inserted between the ATG and the T7 promoter transcription start site, respectively. We transfected the plasmid into HeLa cells and then infected them with a recombinant VACV, vT7LacOi, which encodes a T7 polymerase gene whose expression is induced by Isopropyl β-D-1-thiogalactopyranoside (IPTG) [23]. Because both plasmids were transfected into VACV-infected cells, the effect of VACV infection on plasmid replication was similar. Nevertheless, when cells were treated with IPTG, significantly more GFP was expressed from the plasmid containing the A-tract (Fig 6A). Small amount of eGFP protein expression was observed without IPTG induction, suggesting highly efficient translation of residual amount of mRNA from leaky expression in this inducible system. We quantified the eGFP mRNA levels by qRT-PCR and found similar mRNA amounts from A-tract-containing plasmid at 5 μM of IPTG induction to that from Kozak sequence-containing plasmid at 25 μM of IPTG (Fig 6B), while the protein level from A-tract-containing plasmid was much higher (Fig 6A).

To establish further the role of the 5’-poly(A) leader in enhancing the translational efficiency of VACV post-replicative mRNA, we made two recombinant viruses expressing IPTG inducible eGFP based on the parental virus vT7LacOi. In the recombinant viruses, the T7 promoter was followed by a Kozak sequence (vT7LacOi_Kozak-GFP) or an A-tract containing 12 residues (vT7LacOi_A12-GFP) driving the eGFP ORF. With increasing concentrations of IPTG, both recombinant viruses responded with increasing GFP expression (Fig 6C). Notably, the levels of eGFP expression were strikingly higher in vT7LacOi_A12-GFP-infected cells (Fig 6C). Again, small amount of eGFP protein expression was observed without IPTG induction, suggesting efficient translation of mRNA from leaky transcription. qRT-PCR showed vT7LacOi_Kozak-GFP infected cells expressed more GFP mRNA, from at 100 and 1000 μM, compared to that from vT7LacOi_A12-GFP-infected cells at 5 μM IPTG induction; however, the protein level was much higher from the latter vT7LacOi_A12-GFP-infected cells at 5 μM (Fig 6D). We further compared the ratio of ribosome-bound GFP mRNA to total GFP mRNA with or without a poly(A) leader in VACV-infected cells by taking advantage of the two recombinant viruses. The result indicated that the relative ratio of the GFP mRNA transcribed from the vT7LacOi_A12-GFP genome (with a poly(A) leader) was significant higher (~3-fold) than that from the vT7LacOi_Kozak-GFP genome (without a poly(A) leader) (Fig 6E). In contrast, the ratios were similar for F17 mRNA, a late viral transcript with 5’-poly(A) leader from both viruses (Fig 6E). Taken together, these data supported that a poly(A) leader rendered VACV post-replicative mRNAs a translational advantage.

### Messenger RNA with a 5’-poly(A) leader capped by an ApppG cap analog can confer a translational advantage in VACV-infected cells

**Fig 7:**
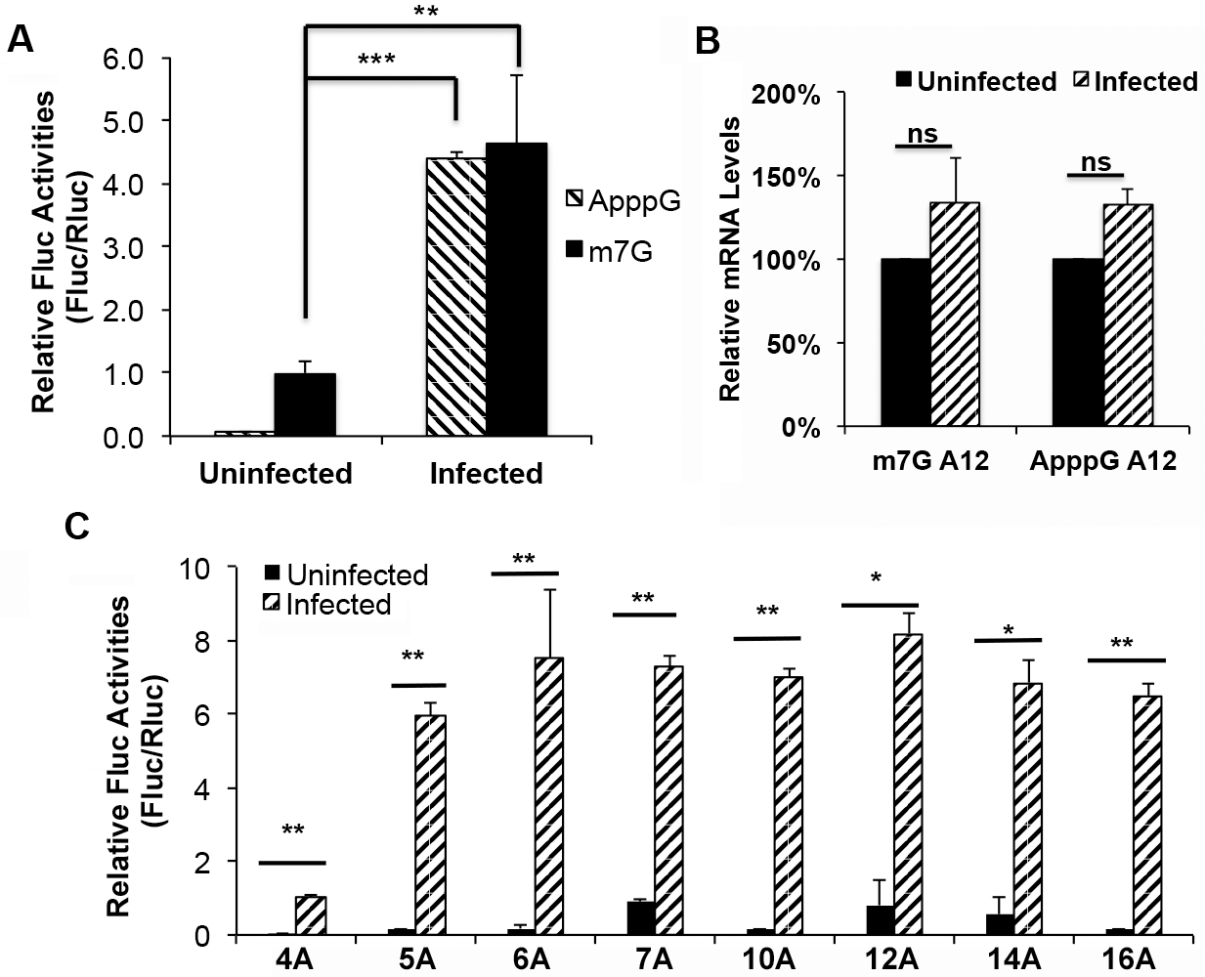
Messenger RNA with a 5’-poly(A) leader capped by ApppG cap analog can confer the translational advantage in VACV-infected cells. (A) Relative luciferase activity from Fluc mRNA with a 5’-poly(A) leader capped with ApppG or m^7^G was measured at 5 h after transfection into uninfected or VACV-infected HeLa cells. Fluc activity was normalized by a co-transfected Rluc mRNA. Fluc activity from m^7^G-capped mRNA in uninfected cells was normalized as 1. (B) Quantitative RT-PCR (qRT-PCR) compared the relative levels of Fluc mRNA capped by ApppG or m^7^G from uninfected and VACV-infected HeLa cells at 5 h post transfection. The amount of mRNA in uninfected cells was normalized as 1. (C) ApppG-capped Fluc reporter mRNAs, with different lengths of 5’-poly(A) leaders, were transfected into uninfected and VACV-infected HeLa cells together with an m^7^G Rluc mRNA. Luciferase activities were measured at 5 h post transfection. Error bars represent standard deviation (SD) of at least three experiments. P-values were determined using the Student’s t-test; ***P value < 0.001, **P value < 0.01, *P value < 0.05, ns = Not Significant (i.e. P value > 0.05).

Only approximately 5 to 10% of the mRNA expressed from the bacteriophage T7 promoter in the VACV expression system contains the m^7^G cap structure [24], suggesting a possibility that the VACV post-replicative mRNAs with 5’-poly(A) leaders can be translated in a cap-independent manner in the experiment of Fig 6C. An ApppG cap cannot initiate cap-dependent translation but does protect mRNA from degradation and so is often used to test cap-independent mRNA translation [25, 26]. We compared Fluc mRNA synthesized with an ApppG-cap to those with an m^7^G-cap, both with a 5’-poly(A) leader. Equal amounts of the mRNAs were transfected into VACV-infected HeLa cells, together with an m^7^G-capped Rluc mRNA, and luciferase activities were measured at 5 h post transfection. As expected, in uninfected cells, a 100-fold higher luciferase activity was produced from the m^7^G-capped mRNA compared to the ApppG-cap mRNA. Strikingly however, in VACV-infected cells, mRNAs capped with an ApppG were also efficiently translated (Fig 7A). The levels of ApppG-capped, as well as the m^7^G-capped mRNA were similar at 5 h post transfection in uninfected and VACV-infected cells (Fig 7B), ruling out the possibility of difference in mRNA levels as a source of different luciferase activities. The translational advantage of ApppG-capped mRNA in VACV-infected cells was also observed in different cell types (S5 Fig). We tested ApppG-capped mRNAs with different lengths of 5’-poly(A) leaders and observed much higher luciferase activity in VACV-infected cells compared to uninfected cells (Fig 7C). Interestingly, the length dependence in ApppG-capped mRNA was different from m^7^G-capped mRNA, which is likely due to different translation factors used in ApppG-capped and m^7^G-capped mRNA translation. In addition, large variations of ApppG-capped RNA translation in uninfected cells also contributed to the difference. Nevertheless, the conclusion of length dependence should be based on the mRNAs with m^7^G-capped mRNAs as the viral mRNAs are capped in VACV infected cells. These results suggested that mRNA with a 5’-poly(A) leader capped by an ApppG cap analog could confer a translational advantage in VACV-infected cells.

### Messenger RNA with a 5’-poly(A) leader can confer a translational advantage in cells with impaired cap-dependent translation

**Fig 8:**
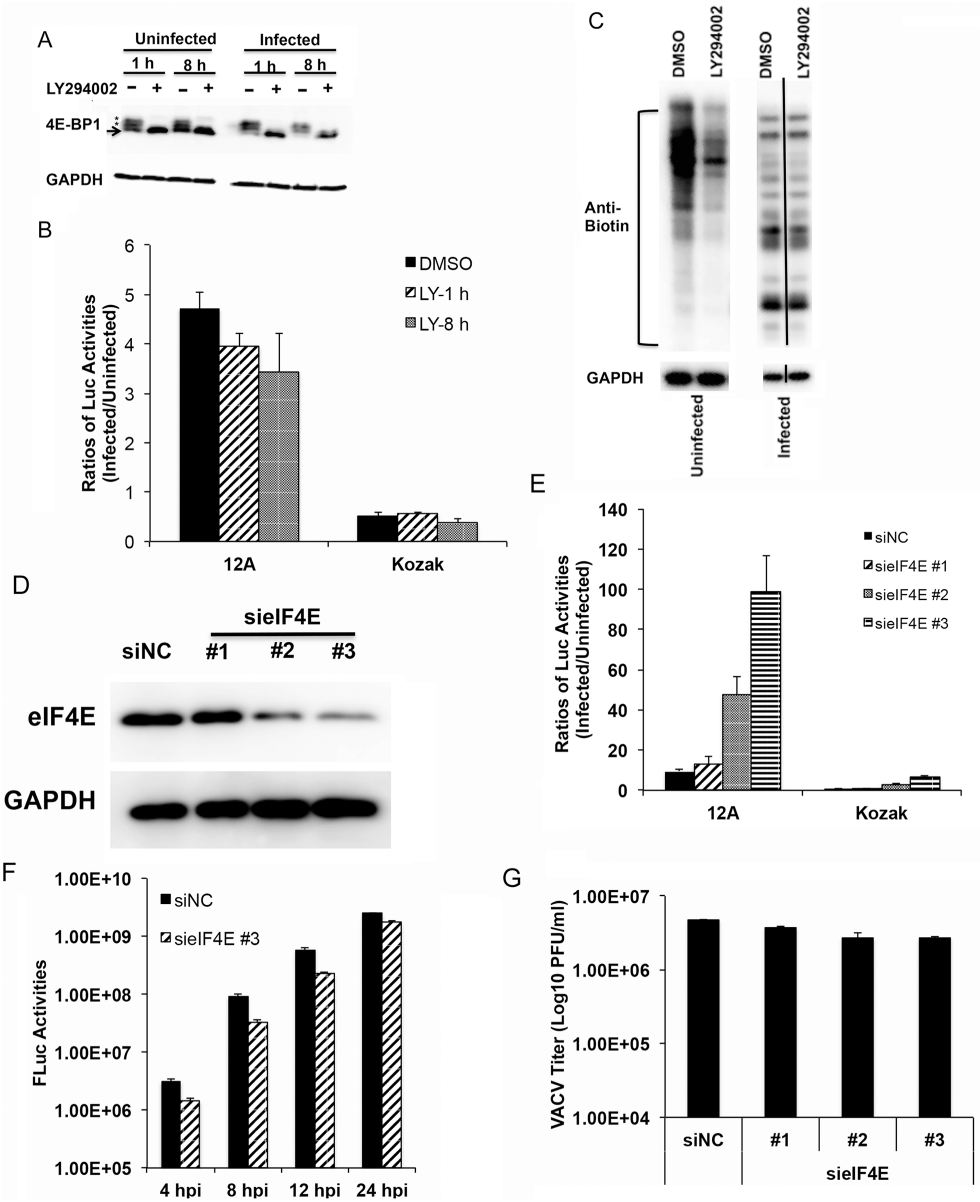
VACV post-replicative mRNAs are efficiently translated when eIF4E is inhibited. (A) Hypophosphorylation of 4E-BP1 was induced by LY294002 in uninfected and VACV-infected HeLa cells at 1 hpi or 8 hpi and the cells were harvested for Western blotting analysis probed using anti-4E-BP1 antibody. An asterisk indicates hyperphosphorylated 4E-BP1. The arrow indicates hypophosphorylated 4E-BP1. (B) HeLa cells were treated with LY294002 at 1 or 8 hpi. An Fluc reporter mRNA with a 12A leader was transfected into uninfected and VACV-infected cells together with an Rluc mRNA at 12 hpi. Luciferase activities were measured at 5 h post transfection. The ratios of Fluc and Rluc activities from VACV-infected to uninfected HeLa cells were calculated, respectively. (C) HeLa cells infected with VACV and treated with LY294002 at 8 hpi. Newly synthesized proteins were labeled by AHA at 20 hpi and detected using antibodies against biotin conjugated to AHA using the Click-IT chemistry-based technique. The blots of infected-cell lysates were from different lanes on the same gel. (D) HeLa cells were transfected with control (siNC) or siRNAs targeting eIF4E. The protein levels of eIF4E and GAPDH were detected using specific antibodies 48 h post transfection. (E) HeLa cells were transfected with control or eIF4E siRNAs as in (D). An Fluc reporter mRNA with a 12A leader was transfected into uninfected and VACV-infected cells together with an Rluc mRNA at 12 hpi. Luciferase activities were measured at 5 h post transfection. The ratios of Fluc and Rluc activities from VACV-infected to uninfected HeLa cells were calculated, respectively. (F) HeLa cells were transfected with control or eIF4E siRNAs as in (D). 48 h post-transfection, the cells were infected with a recombinant VACV expressing firefly luciferase gene under a viral early/late promoter. Luciferase activities were detected at 8 hpi. (G) HeLa cells were transfected with control or eIF4E siRNAs as in (D). 48 h post-transfection, the cells were infected with VACV. The viral titers were determined at 24 hpi using a plaque assay. Error bars represent standard deviation (SD) of at least three experiments. P-values were determined using the Student’s t-test; **P value < 0.01.

Cap-dependent translation depends on the eukaryotic translation initiation factor 4E (eIF4E), which binds the 5’ cap (m^7^G) and recruits other translation-initiation components. The eIF4E binding protein 1 (4E-BP1) can bind to eIF4E and inhibit translation initiation; and 4E-BP1 hyperphosphorylation disassociates it from eIF4E, allowing translation to initiate [27]. To inhibit 4E-BP1 hyperphosphorylation, we suppressed PI3-kinase with the small molecule, LY294002, at 1 hpi and 8 hpi [28] (Fig 8A). The treatment reduced luciferase activities from transfected Fluc mRNA (with a poly(A) leader) and Rluc mRNA (with a 5’-UTR containing a Kozak sequence) in uninfected cells (S6A Fig). In VACV-infected cells, mRNA with a 5’-poly(A) leader was still more efficiently translated, although somewhat less than in untreated cells, indicated by the ratios (3–5 fold) of firefly luciferase activities from VACV-infected cells to that from mock-infected cells (Fig 8B). In contrast, the corresponding ratios of renilla luciferase activities from co-transfected mRNA containing a Kozak element in its 5’-UTR were lower than one (Fig 8B). It is worth noting that we did not use renilla luciferase activities to normalize the firefly luciferase activities as the impairment of cap-dependent translation presumably affected renilla mRNA translation. Moreover, in VACV-infected HeLa cells treated with LY294002 from 8 hpi, nascent viral protein synthesis was only slightly inhibited by LY294002, whereas translation of cellular proteins in uninfected cells was noticeably lower (Fig 8C). The treatment at 8 hpi rather than at earlier time of infection was to reduce the impact of unphosphorylated 4E-BP1 on VACV replication as early treatment of cells with LY294002 could inhibit VACV replication [28].

We employed another approach to impair cap-dependent translation by using three specific siRNAs that knocked down eIF4E at various levels in HeLa cells (Fig 8D). The knockdown of eIF4E reduced luciferase activities from transfected Fluc mRNA (with a poly(A) leader) and Rluc mRNA (with a 5’-UTR containing a Kozak sequence) in uninfected cells, corresponding to the levels of eIF4E knockdown (S6B Fig). In contrast, in VACV-infected cells with eIF4E knockdown, the mRNA containing a poly(A) leader was efficiently translated, indicated by the high ratios of firefly luciferase activities from VACV-infected cells to that from uninfected cells (Fig 8E). The corresponding ratios of renilla luciferase activities from co-transfected mRNA containing a Kozak element in its 5’-UTR were much lower (Fig 8E), suggesting selective translation of poly(A)-headed mRNA in eIF4E knockdown cells infected with VACV. Consequently, the knockdown of eIF4E only reduced VACV gene expression at late time by approximately 2-fold using a reporter VACV expression firefly luciferase gene under the control of an early/late promoter [29] (Fig 8F). Similarly, there was only less than 2-fold reduction of virion production by a plaque assay (Fig 8G). Together, these findings demonstrated that even if cap-dependent translation was inhibited, mRNA with a 5’-poly(A) leader was still efficiently translated during the post-replicative stage of VACV replication.

### The 5’-poly(A) leader is not an internal ribosome entry site (IRES)

**Fig 9:**
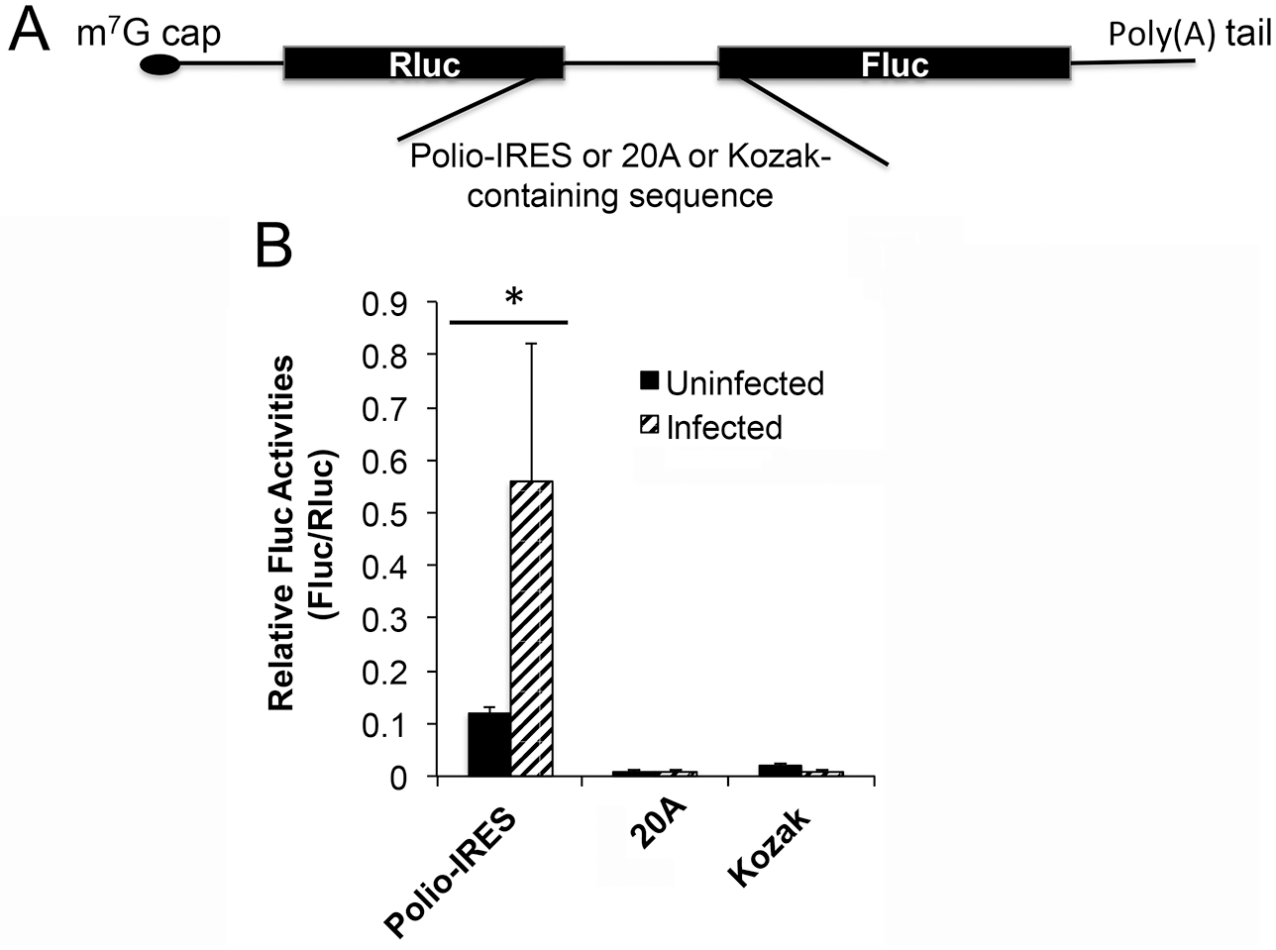
The 5’-poly(A) leader is not an internal ribosome entry site (IRES). (A) Schematic of the bicistronic reporter mRNAs flanked by a poliovirus IRES, 20 A residues, or a Kozak-containing sequence. (B) The in vitro transcribed bicistronic mRNA was transfected into uninfected and VACV-infected HeLa cells and luciferase activities were measured 5 h post transfection. The ratios of Fluc to Rluc activities were calculated and displayed. Error bars represent standard deviation (SD) of at least three experiments. P-values were determined using the Student’s t-test; *P value < 0.05, ns = Not Significant (i.e. P value > 0.05).

The IRES is the best-studied cap-independent mechanism and is used by many RNA viruses, e.g., poliovirus uses an IRES to initiate cap-independent translation [30, 31]. To test whether an A-tract can function as an IRES, we synthesized a bicistronic mRNA in which renilla luciferase (Rluc) and firefly luciferase (Fluc) coding sequences are connected by a poliovirus IRES (polio IRES), an A-tract with 20 A residues, or a Kozak-containing 20-nt sequence (Fig 9A). The synthetic mRNA had an m^7^G cap so Rluc translation was cap-dependent. The polio IRES should drive Fluc translation whereas the Kozak-containing sequence should not. Since our reporter system was based on mRNA rather than a plasmid we could rule out interference from unexpected Fluc transcripts driven by cap-dependent translation. Synthetic RNA was transfected into uninfected or VACV-infected HeLa cells and the Fluc and Rluc activities were measured. As expected, Fluc driven by a poliovirus IRES was translated considerably in both uninfected and VACV-infected cells (Fig 9B); however, Fluc expression driven by the A-tract was negligible in both uninfected and VACV-infected cells, and at a level similar to that from the Kozak-containing sequence (Fig 9B). Interestingly, a comparison of Fluc and Rluc activities in uninfected and VACV infected cells indicated that the poliovirus IRES-driven Fluc mRNA translation was enhanced in VACV-infected cells, as evidenced by the significantly higher Fluc/Rluc ratio of the poliovirus IRES bicistronic mRNA (Fig 9B). These data indicated that the poly(A) leader did not function as an IRES.

## Discussion

### Conferring a translational advantage by the 5’-poly(A) leader during poxvirus infection

After infection, VACV takes over host cell machineries to synthesize viral proteins rapidly and shut off cellular protein synthesis globally [32, 33]. This can be largely attributed to cellular mRNA depletion and production of a large amount of viral mRNAs [11, 22]. Cellular mRNA decay is accelerated due to VACV-encoded decapping enzymes and cellular transcription is also inhibited [34–39]. In the meantime, virus-encoded, DNA-dependent RNA polymerase efficiently transcribes VACV mRNAs. Several hours after infection of HeLa cells, 70% of total mRNA is viral [11, 12]. In VACV-infected cells, although the role of mRNA manipulation in host protein synthesis shutoff seems clear, it remains largely elusive whether and how the translational control contributes. As the takeover of protein synthesis in infected cells occurs after VACV DNA replication, it is conceivable that the VACV post-replicative mRNAs exert a translational advantage.

The 5’-poly(A) leader in several post-replicative VACV mRNAs was initially discovered almost three decades ago [6–8, 13]. Our genome-wide survey demonstrated that all viral post-replicative mRNAs contain 5’-poly(A) leaders [5]. The 5’-UTR can regulate translation efficiency of an mRNA [9]. Nevertheless, it was unclear whether the 5’-poly(A) leader has a specific function or is simply because the VACV RNA polymerase “accidentally” slips during transcription at three T residues on the template strand. This study demonstrated that poxviruses use the 5’-poly(A) leader to efficiently synthesize viral post-replicative proteins, most of which are the viral building blocks. The finding here demonstrated the role of the 5’-poly(A) leader in efficient translation of VACV post-replicative mRNAs during VACV replication. However, our findings do not rule out other mechanisms that confer a translational advantage of VACV post-replicative mRNAs. Interestingly, although the enhancement of poly(A)-headed mRNA in VACV-infected cells in all experiments, the enhancement levels varied in different experiments. The variations were likely due to quick VACV replication that resulted in variations of cellular environments at the time of RNA transfection. Most of the 5’-poly(A) leaders of VACV post-replicative mRNAs are between 8 and 12 A residues [5]; this agrees with data showing 5’-poly(A) leaders within that range confer the optimal translational advantage. Thus, the 5’-poly(A) leader is convincingly an evolutionarily optimized, transcriptionally and translationally coordinated element that VACV utilizes to maximize its protein production.

A-rich tracts with various lengths are present in other eukaryotic mRNA 5’-UTRs. In yeast, A-rich tracts can be found in over 3,000 5’-UTRs. Interestingly in yeast, protein abundance correlates with the size of the A-rich tracts and mRNAs containing 5’-UTRs with 12 consecutive A residues generate large amounts of protein. Although the number of A residues is similar to those in the 5’-poly(A) leaders of VACV mRNAs, the yeast A-tracts are usually embedded internally in the 5’-UTR [40]. In contrast, poly(A) leaders of VACV mRNAs are located at the very 5’ end and usually are the only 5’-UTR sequence. An A-rich tract was also found in the 5’-UTRs of other viruses, such as crucifer-infecting tobamovirus and avian herpesvirus [41, 42]; however, as in yeast, they are usually present inside of the 5’-UTRs rather than at the very 5’ end. Additionally, unlike many of the other A-rich tracts, the 5’-poly(A) leader of VACV mRNA is not an IRES, although it can be efficiently translated in cells with impaired cap-dependent translation. These differences suggest poxvirus mRNAs employ a distinct mechanism.

Although our findings strongly indicate higher translation efficiency of poly(A)-headed mRNA in VACV-infected cells, especially since there was no significant difference with transfected RNAs after 5 h, the possibility that poly(A)-headed mRNA transcribed from viral genome is more stable in VACV-infected cells has not been ruled out. In fact, we observed higher levels of 12A-headed mRNA (IPTG at 5 μM) than Kozak sequence-headed mRNA (IPTG at 25 μM) transcribed from recombinant VACV genome (Fig 6D), which could be attributed to higher mRNA stability and/or more active transcription of the 12A-headed mRNA.

### Could the 5’-poly(A) leader mediate cap-independent translation during poxvirus infection?

Many RNA viruses, such as piconaviruses, crucifer-infecting tobamovirus, hepatitis C virus, and Foot-and-mouth disease virus can synthesize their proteins through a cap-independent translation mode [41, 43–45]. The most studied mechanism is through viral IRESs, which usually bear highly complex structures to recruit 40S ribosome [46]. Some RNA viruses use other cap-independent mechanism such as 3’ cap-independent translational enhancer (3’CITE) in their mRNAs to recruit ribosome subunits [47, 48]. Cap-independent translation is less appreciated in DNA viruses. In this study, we show that a poly(A)-headed mRNA is efficiently translated in cells with impaired cap-dependent translation (Fig 8) as well as without an m^7^G cap (Fig 7), which strongly suggests that a short, unstructured 5’-poly(A) leader may mediate cap-independent translation in VACV-infected cells. In literature, Mulder et al. showed that VACV protein synthesis only requires a low level of intact translation initiation factor eIF4F [49]. In another in vitro study, Shirokikh et al. showed that the translation initiation complex could be formed on a 5’-poly(A) leader mRNA without the need of eIF4E, a rate-limiting and cap binding translation factor [50]. Additionally, in yeast, crucifer-infecting tobamovirus, and avian herpesvirus, an A-rich tract in some 5’-UTRs is suggested to function as an IRES [26, 41, 42], an RNA element allowing for a form of 5’ cap-independent translation initiation. Recruitment of poly(A) binding protein (PABP) through the A-rich tracts plays an important role in IRES-mediated cap-independent translation initiation. During the review process of this manuscript, Jha et al. reported that a small ribosomal subunit protein, receptor for activated C kinase (RACK1), is important for efficient translation of VACV post-replicative mRNAs [51]. RACK1 was previously found to be important for IRES-mediated cap-independent translation of several RNA viruses [52]. These findings support the possibility that the poly(A) can mediate cap-independent translation although it does not serve as an IRES (Fig 9).

VACV encodes its own capping enzymes, which cap VACV mRNAs with methylated guanosine, including the post-replicative mRNAs [15]. The mRNA translation occurs in the “viral factory”, the site of viral replication [53]. Cap-dependent translation initiation factors are recruited to and concentrated within discrete subcellular compartments of the viral factory [54]. VACV mRNAs can be translated in a cap-dependent manner. The possibilty that a cap-independent translation mode can be employed by the VACV post-replicative mRNAs to confer a translational advantage does not exclude cap-dependent translation used by these mRNAs. It is likely that VACV uses both cap-independent and cap-dependent translation modes to maximize the translation potential of viral post-replicative mRNAs in different cellular environments. VACV infection globally shuts off host protein synthesis after DNA replication, coninciding with viral post-replicative mRNA synthesis [32, 33]. During this stage, it is conceiveable that the ability to utilize cap-independent translation is important because the global protein synthesis shutoff downregulates expression of most host proteins, including cap-dependent translation initiation factors. Meanwhile, a large amount of viral mRNAs need to be translated. Cap-independent translation could also ensure efficient viral mRNA translation in other physiological conditions, such as during cell mitosis and various stress conditions (including poxvirus infection itself), in which cap-dependent translation is suppressed [55]. In fact, under different conditions, some eukaryotic cellular mRNAs can employ both translational modes to ensure synthesis of necessary corresponding proteins [46]. Similar to these cellular mRNAs, the VACV post-replicative mRNAs may switch between cap-dependent and cap-independent translation according to the availability of eukaryotic translation initiation factors and cellular environments.

While the experiments using an ApppG-capped RNA strongly support a cap-independent translation mode during the post-replicative stage of VACV replication (Fig 7), a caveat of the experiments is that VACV encodes both decapping and recapping enzymes that may remove and recap the transfected ApppG-capped mRNA in VACV-infected cells [14, 34, 35, 56]. We do not rule out the possibility that the poly(A)-headed mRNA employs an alternative cap-dependent translation mode that requires a minimal amount of cap-binding translation initiation factor eIF4E. In fact, a minimal requirement of the eIF4F translation initiation complex has also been observed during the late stage of cytomegalovirus infection [57]. Future experiments will further investigate these aforementioned different possibilities. Recombinant VACVs with defective decapping or capping enzyme expression will be useful in such study.

As all viruses rely on host translation machinery to synthesize viral proteins, VACV likely employs and modulates an existing cellular mechanism for its mRNA translation. The data presented here is an important step in uncovering this novel cellular translation mechanism. The 5’-poly(A) leader can also be used to increase foreign gene expression when using poxvirus-based vectors, as demonstrated in this study.

## Materials and methods

### Cells and viruses

HeLa cells (ATCC-CCL2) and human foreskin fibroblasts (HFFs, kindly provided by Dr. Nicholas Wallace) were cultured in Dulbecco’s modified eagle’s medium (DMEM, Quality Biological) with 10% fetal bovine serum (FBS, Peak Serum). BHK21 [C13] (ATCC CCL10), RK13 (ATCC CCL37) and BS-C-1 (ATCC CCL-26) cells were cultured in Eagle's Minimum Essential Medium (EMEM, Quality Biological) with 10% FBS (Peak Serum). All cells were incubated in a 5% CO_2_ atmosphere at 37°C. The recombinant VACV with a firefly luciferase gene under the control of a viral early/late promoter was a gift from Dr. Bernard Moss and described previously [29]. The recombinant VACV with intermediate transcription factor gene A23 deletion (vA23Δ) was a gift from Dr. Bernard Moss and was described elsewhere [20]. Preparation, infection and titration (by plaque assay) of the VACV Western Reserve (WR) strain (ATCC VR-1354) and recombinant VACVs derived from it were carried out as described elsewhere [58]. Myxoma virus was kindly provided by Dr. Stefan Rothenburg.

### In vitro RNA synthesis

Primers were designed to produce DNA fragment containing the T7 promoter followed by a 5’-UTR sequence of interest, reporter gene (firefly or renilla Luciferase) and poly(A) tail coding sequence. These primers were used to synthesize DNA fragments by PCR using a Q5 High-Fidelity 2X Master Mix (New England Biolabs). The synthesized DNA was used as template to generate RNA using a HiScribe T7 Quick High Yield RNA Synthesis Kit (New England Biolabs). The synthesized RNA was capped using m^7^G (Anti-Reverse Cap Analog [ARCA]) or ApppG cap analog (New England Biolabs) according to the manufacturer’s instructions. The resulting RNA was purified using a PureLink RNA Mini Kit (Thermo Fisher Scientific) and quantified using a NanoDrop ND-2000 instrument (Thermo Fisher Scientific). The IRES reporter plasmid pcDNA3 RLUC POLIRES FLUC used for biscistronic mRNA production was a gift from Nahum Sonenberg (Addgene plasmid # 45642) [31].

### Transfection and luciferase assay

Capped and polyadenylated firefly luciferase RNA (480 ng in one well of a 24-well plate) was transfected into cells using Lipofectamine 2000 Transfection Reagent (Thermo Fisher Scientific) according to the manufacturer’s instructions. When necessary, renilla luciferase reporter RNA (20ng in one well of a 24-well plate) was co-transfected as an internal control. The luciferase activities were measured at 5 h post transfection using a dual-Luciferase Reporter Assay System (Promega), according to the manufacturer’s instructions, on a microplate luminometer (Promega).

### Knockdown of eIF4E using small interfering RNA (siRNA)

The siRNAs were purchased from Integrated DNA technologies (IDTDNA). HeLa cells were transfected with sieIF4E or siNC (negative control) at the final concentration of 5 nM using Lipofectamine RNAiMax (Thermo Fisher Scientific) according to the manufacturer's instructions. The cells were harvested to examine the effects of siRNA knockdown by Western blotting analysis or infected with VACV for various assays 48 h post transfection.

### Western blotting analysis

Cells were lysed and heated in sample buffer. The cell lysates were resolved by sodium dodecyl sulfate-polyacrylamide gel electrophoresis (SDS-PAGE). The proteins were transferred onto a polyvinylidene difluoride membrane, which was blocked with 5% bovine serum albumin (BSA) in TBST solution (50 mM Tris-HCl [pH 7.5], 200 mM NaCl, 0.05% Tween 20) at room temperature for 1 h. The membrane was then incubated with primary antibody in TBST-BSA buffer for 1 h at room temperature or overnight at 4°C, washed with TBST, incubated with horseradish peroxidase-conjugated secondary antibody for 1 h at room temperature, washed with TBST, and developed using a chemiluminescent substrate. The intensities of the bands were quantified using Image J.

### Antibodies and chemicals

Anti-GFP, anti-eIF4E and anti-4E-BP1 antibodies were purchased from Cell Signaling Technology. Anti-GAPDH was purchased from Abcam. LY294002 was purchased from Cayman Chemical.

### Pulse-chase labeling and detection of newly synthesized proteins with AHA (L-azidohomoalanine)

The experiment was carried out using Click-iT AHA nascent protein kit (Thermo Fisher Scientific). Briefly, HeLa cells were cultured in a T-75 flask to a confluence of 95%. The cells were infected or mock infected with VACV at a MOI of 10. The cells were treated with DMSO or LY294002 (25 μM) at the desired times of infection. Cell culture medium was replaced with methionine-free medium at the indicated times. After incubation in methionine-free medium, AHA was added to the medium at 100 μM for 2 h. The cells were scraped off the flask and collected by centrifuging. Cell pellets were then suspended with 500 μl of lysis buffer containing 500U of benzonase for 30 min. After centrifuging at 12,000 g at 4°C for 10 min. The proteins were precipitated with methanol and chloroform and resolubilized in 50 mM Tris-HCl containing 1% SDS, pH 8.0. AHA-containing peptides were labeled with alkyne-biotin, by subjecting 200 μg of proteins to the click reaction for 30 min using the Click-iT protein labeling kit according to the manufacturer’s instruction (Thermo Fisher Scientific). Proteins were re-precipitated with methanol and chloroform, solubilized with 50mM Tris-HCl containing 1% SDS, pH 8.0, for Western blotting analysis.

### Recombinant VACV generation

The recombinant viruses used in this study include vT7LacOi-Kozak-GFP and vT7LacOi-A12-GFP. The vT7LacOi-Kozak-GFP and vT7LacOi-A12-GFP were derived from the parental virus vT7LacOi that is capable of isopropyl-beta-D-thiogalactopyranoside (IPTG)-inducible T7 promoter-controlled expression of foreign genes [59]. The eGFP-encoding sequence downstream of either a Kozak encoding sequence (A ATT GTG AGC GCT CAC AAT TCC CGC CGC CAC C; vT7LacOi-Kozak-GFP) or 12 A residues (vT7LacOi-A12-GFP), under the control of a T7 Promoter, were inserted between the VACWR146 and 147 ORFs.

### Quantitative reverse transcription PCR (qRT-PCR)

Total RNA was extracted using TRIzol reagent (Ambion) followed by purification using a PureLink RNA Mini Kit (Thermo Fisher Scientific). The RNA was used to synthesize cDNA using SuperScript III First-strand synthesis (Invitrogen) according to the manufacturer’s instructions using random hexamers. Quantitative RT-PCR was carried out using iTaq Universal SYBR Green Supermix (Bio-Rad) according to the manufacturer’s directions and specific primers of desired genes.

### Isolation of total RNA and ribosome- and polysome-bound RNA

HeLa cells were treated with 100 μg/ml cycloheximide for 15 minutes at 37°C before harvesting. The harvested cells were processed to isolate total mRNAs or ribosome- and polysome-bound mRNAs as described with modifications [60]. The total RNA was isolated using TRIzol reagent followed by purification using a PureLink RNA Mini Kit. For ribosome/polysome-bound RNA, the harvested cells were resuspended in ribosome homogenization buffer (50 mM Tris-HCl (pH 7.5), 5 mM MgCl_2_, 25 mM KCl, 1% Triton X-100, 100 μg/ml cycloheximide, 10 mM Vanadyl ribonucleoside complex and 0.2 M sucrose) and incubated for 20 min on ice. After centrifuging at 20,000 g for 10 min at 4°C, the supernatant was collected and gently layered over sucrose cushion buffer (50 mM Tris-HCl (pH 7.5), 5 mM MgCl_2_, 25 mM KCl, 100 μg/ml cycloheximide, 10 mM Vanadyl ribonucleoside complex and 2 M sucrose) and ribosome homogenization buffer (1:1 ratio). The sucrose cushion was centrifuged at 35,000 rpm using SW41 Ti rotor for 20 hrs. The ribosome and polysome-bound mRNA was isolated from the pellet using TRIzol reagent followed by purification using a PureLink RNA Mini Kit. DNA was removed using DNase I from RNA samples.

### Relative translation efficiency analysis

Relative translation efficiency (TE) was defined as the ratio of ribosome-protected RNA reads to mRNA reads as described elsewhere [61, 62]. The mRNA and ribosome-protected RNA reads were obtained from the studies described elsewhere [21, 22].

## Supporting information

S1 Fig Fluc and Rluc activities increase over time after transfection into uninfected or VACV-infected HeLa cells. Fluc mRNA with a 5’-poly(A) leader of 20 residues was transfected into uninfected or wild-type VACV-infected HeLa cells (12 hpi) together with an Rluc mRNA with a 5’-UTR containing a Kozak sequence. Luciferase activities were measured at indicated times post transfection. Relative Fluc (A) or Rluc (B) activities from uninfected or VACV-infected cells at different times were displayed. The Rluc or Fluc activities at 1 h post transfection were normalized as 1.
(TIF)

S2 Fig The optimal length of 5’-poly(A) leader in conferring translational advantage is 12 residues. The Fluc reporter mRNAs with different 5’-poly(A)-leaders lengths were transfected into VACV-infected HeLa cells, together with an Rluc mRNA. The FLuc activities were measured at 5 h post transfection and shown in this figure. Error bars represent standard deviation (SD) of at least three experiments.
(TIF)

S3 Fig An mRNA with a 5’-poly(A) leader confers a translational advantage during the post-replicative stage of VACV replication. Fluc mRNA with a 5’-poly(A) leader of 12 residues was transfected into uninfected or wild-type VACV-infected HeLa cells together with an Rluc mRNA at indicated times post infection. Luciferase activities were measured at 5 h post transfection. The Rluc-normalized Fluc activity was normalized as 1 in uninfected HeLa cells.
(TIF)

S4 Fig An uninterrupted 5’-poly(A) leader is essential for optimal translation in VACV-infected cells. Fluc reporter mRNAs containing each of the mutated 5’-poly(A) leaders (mutated to G) were transfected into uninfected or VACV-infected HeLa cells, together with an Rluc mRNA. Luciferase activities were measured at 5 h post transfection. The Rluc normalized Fluc activity was normalized as 1 in uninfected HeLa cells. Error bars represent standard deviation (SD) of at least three experiments.
(TIF)

S5 Fig Messenger RNA with a 5’-poly(A) leader capped by an ApppG cap analog is efficiently translated in different types of VACV-infected cells. ApppG-capped, 12A-headed Fluc reporter mRNA was transfected into indicated uninfected and VACV-infected cells together with an m^7^G-capped Rluc mRNA. Luciferase activities were measured at 5 h post transfection. The Rluc normalized Fluc activities were normalized as 1 in uninfected cells. Error bars represent standard deviation (SD) of at least three experiments.
(TIF)

S6 Fig Translation of m^7^G-capped mRNA decreases in uninfected cells with impaired cap-dependent translation initiation factor eIF4E. (A) HeLa cells were treated with DMSO or LY294002 at indicated times in mock-infected cells. An Fluc reporter mRNA headed with 12 As was transfected into uninfected cells together with an Rluc mRNA with a Kozak sequence-containing 5’-UTR at 12 hpi. Firefly (left) and renilla (right) luciferase activities were measured at 5 h post transfection. Luciferase activities were normalized as 1 in DMSO treated cells. (B) HeLa cells were transfected with control (siNC) or siRNAs targeting eIF4E for 48 h. An Fluc reporter mRNA headed with 12 As was transfected into uninfected cells together with an Rluc mRNA with a Kozak sequence-containing 5’-UTR at 12 hpi. Firefly (left) and renilla (right) luciferase activities were measured at 5 h post transfection. Error bars represent standard deviation (SD) of at least three experiments. Luciferase activities were normalized as 1 in siNC-transfected cells. Error bars represent standard deviation (SD) of at least three experiments.
(TIF)
