# Supplementary figures and images for: The 5'-poly(A) leader of poxvirus mRNA confers a translational advantage that can be achieved in cells with impaired cap-dependent translation
Source: PLoS Pathog. 2017 Aug 30;13(8):e1006602. doi: 10.1371/journal.ppat.1006602 (PMC5595341; doi:10.1371/journal.ppat.1006602)

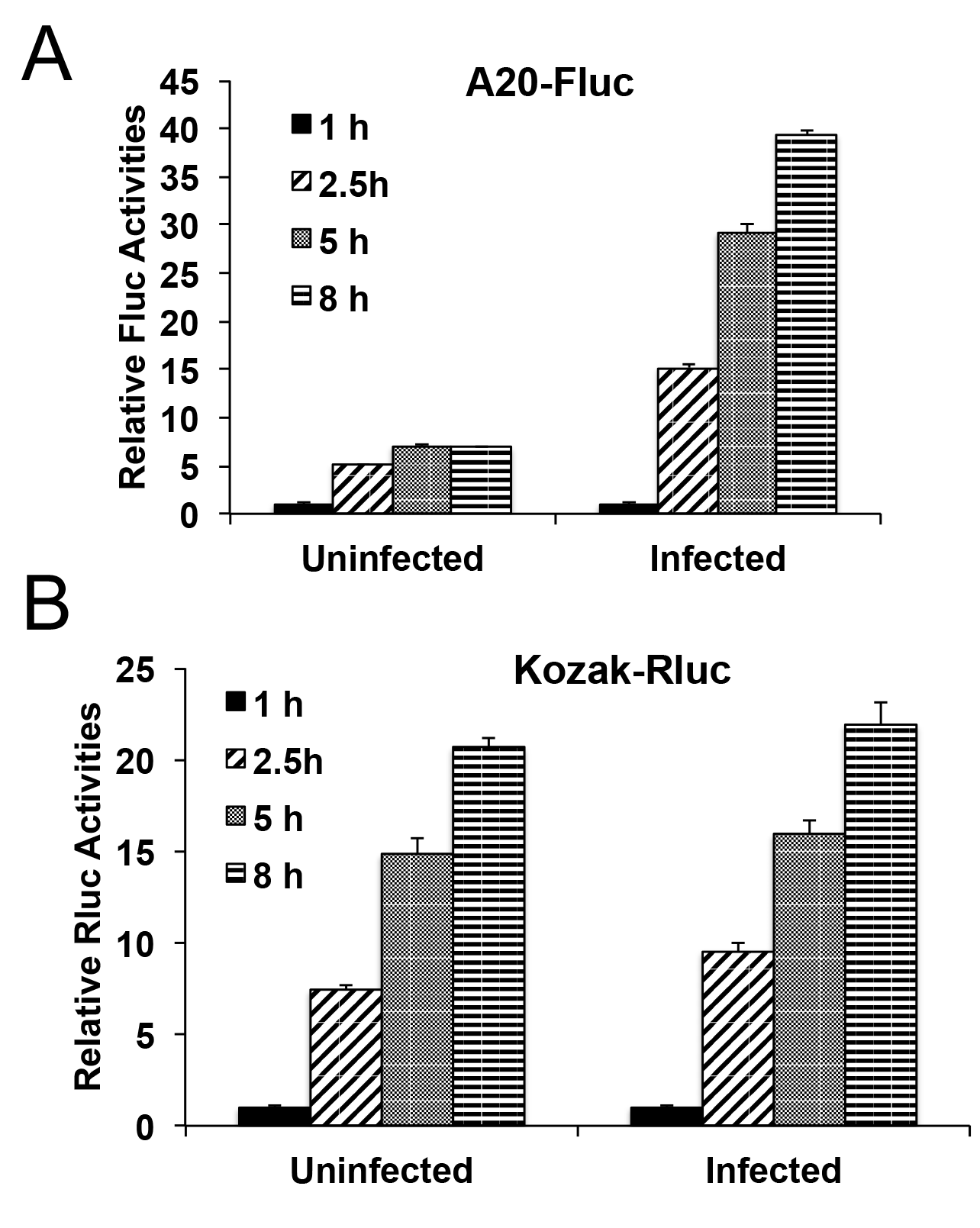

Supplement: S1 Fig — Fluc mRNA with a 5’-poly(A) leader of 20 residues was transfected into uninfected or wild-type VACV-infected HeLa cells (12 hpi) together with an Rluc mRNA with a 5’-UTR containing a Kozak sequence. Luciferase activities were measured at indicated times post transfection. Relative Fluc (A) or Rluc (B) activities from uninfected or VACV-infected cells at different times were displayed. The Rluc or Fluc activities at 1 h post transfection were normalized as 1. (TIF) [file ppat.1006602.s001.tif]

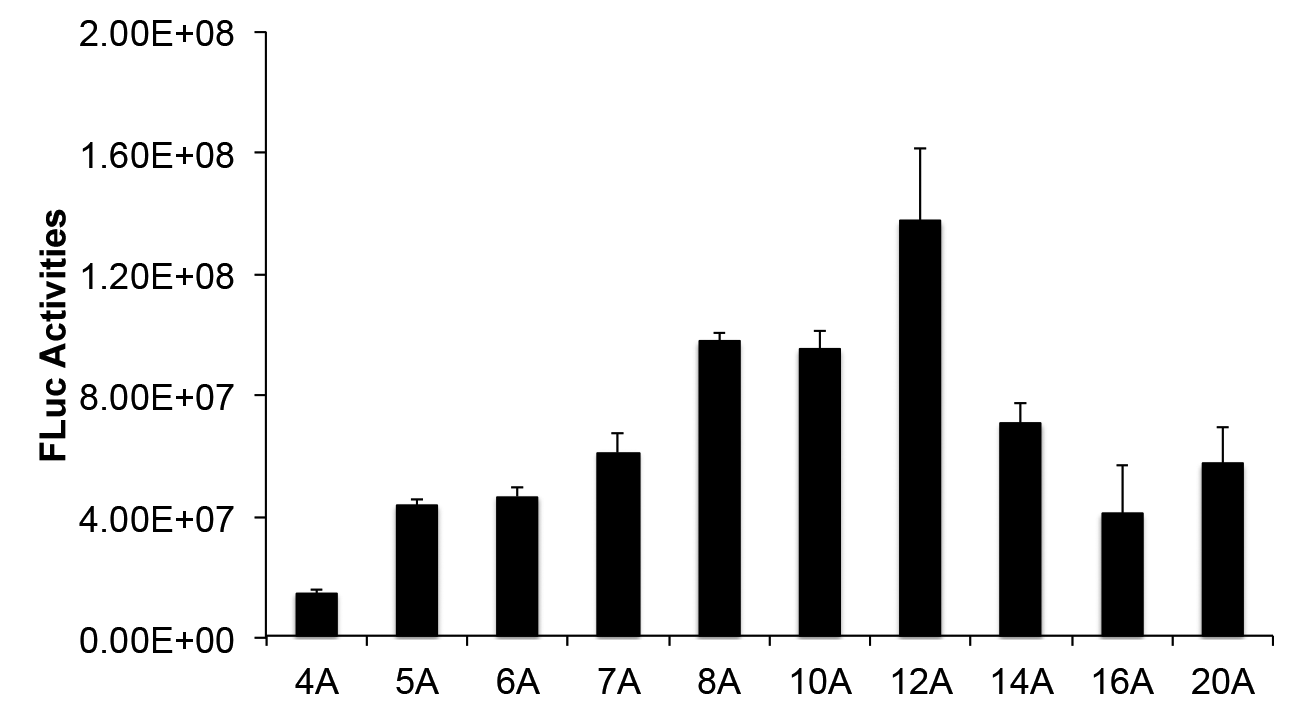

Supplement: S2 Fig — The Fluc reporter mRNAs with different 5’-poly(A)-leaders lengths were transfected into VACV-infected HeLa cells, together with an Rluc mRNA. The FLuc activities were measured at 5 h post transfection and shown in this figure. Error bars represent standard deviation (SD) of at least three experiments. (TIF) [file ppat.1006602.s002.tif]

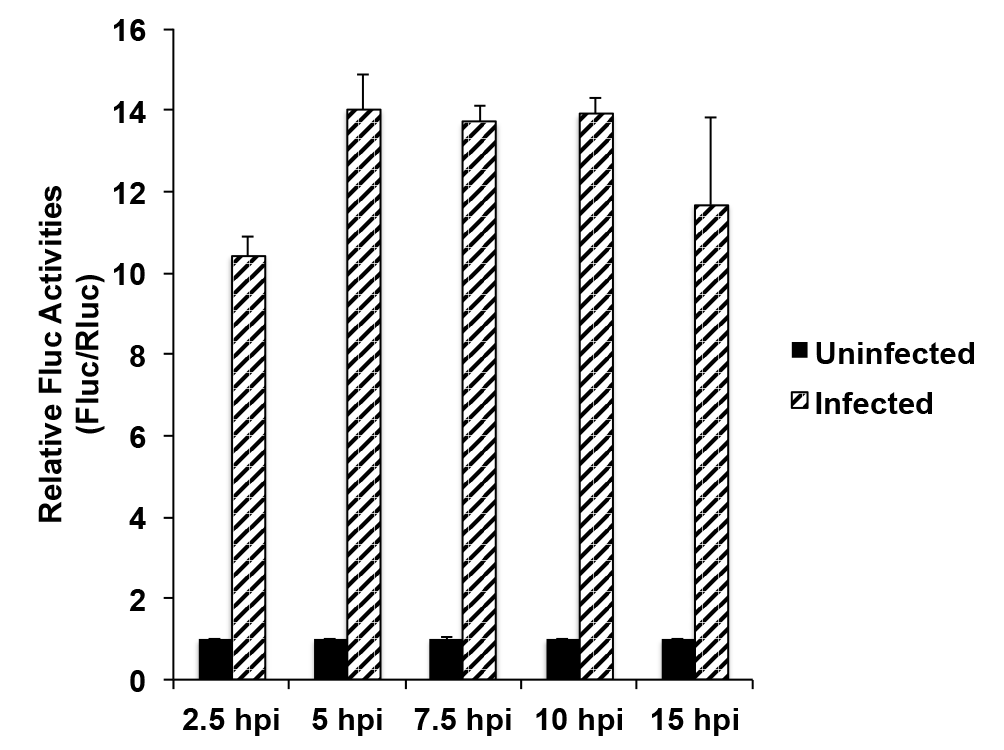

Supplement: S3 Fig — Fluc mRNA with a 5’-poly(A) leader of 12 residues was transfected into uninfected or wild-type VACV-infected HeLa cells together with an Rluc mRNA at indicated times post infection. Luciferase activities were measured at 5 h post transfection. The Rluc-normalized Fluc activity was normalized as 1 in uninfected HeLa cells. (TIF) [file ppat.1006602.s003.tif]

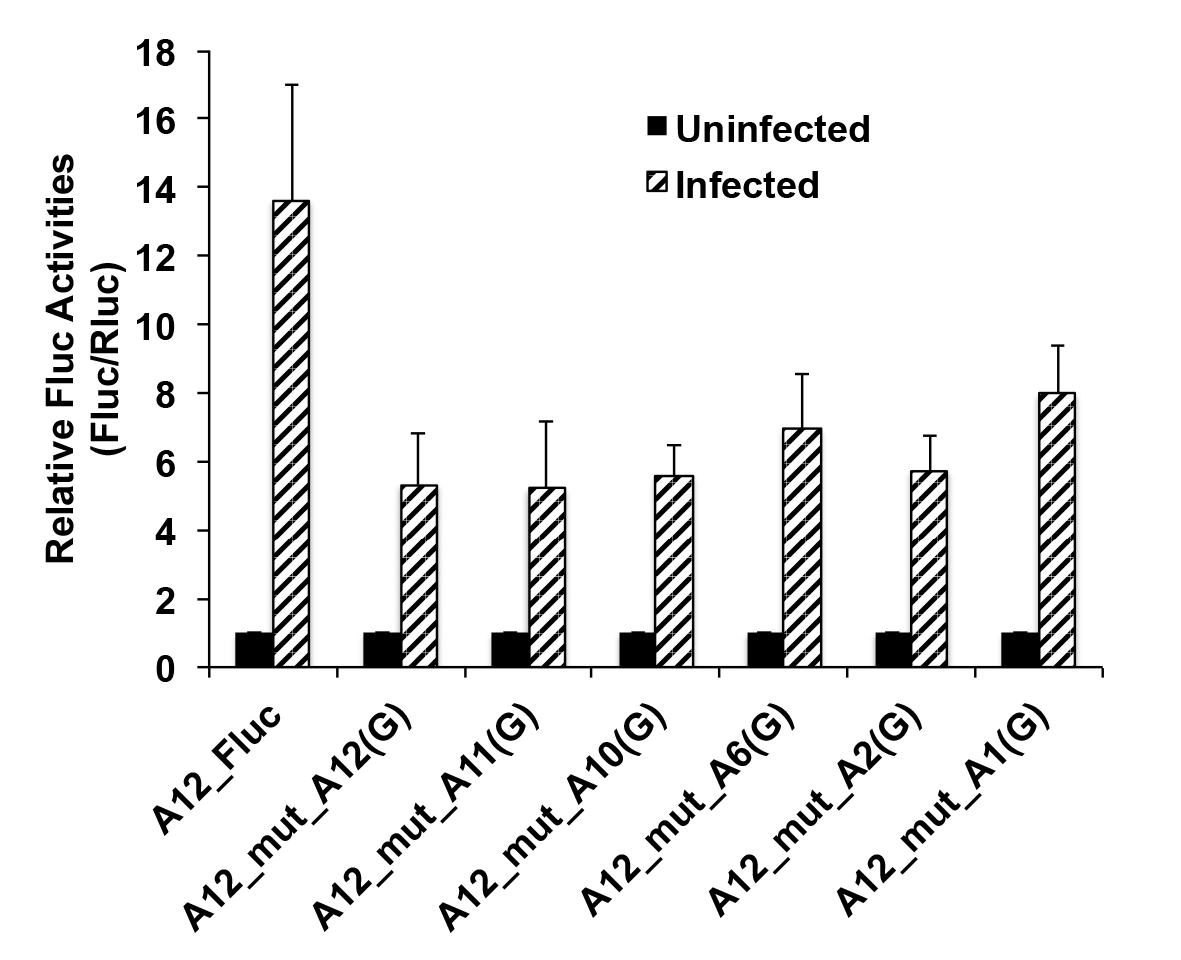

Supplement: S4 Fig — Fluc reporter mRNAs containing each of the mutated 5’-poly(A) leaders (mutated to G) were transfected into uninfected or VACV-infected HeLa cells, together with an Rluc mRNA. Luciferase activities were measured at 5 h post transfection. The Rluc normalized Fluc activity was normalized as 1 in uninfected HeLa cells. Error bars represent standard deviation (SD) of at least three experiments. (TIF) [file ppat.1006602.s004.tif]

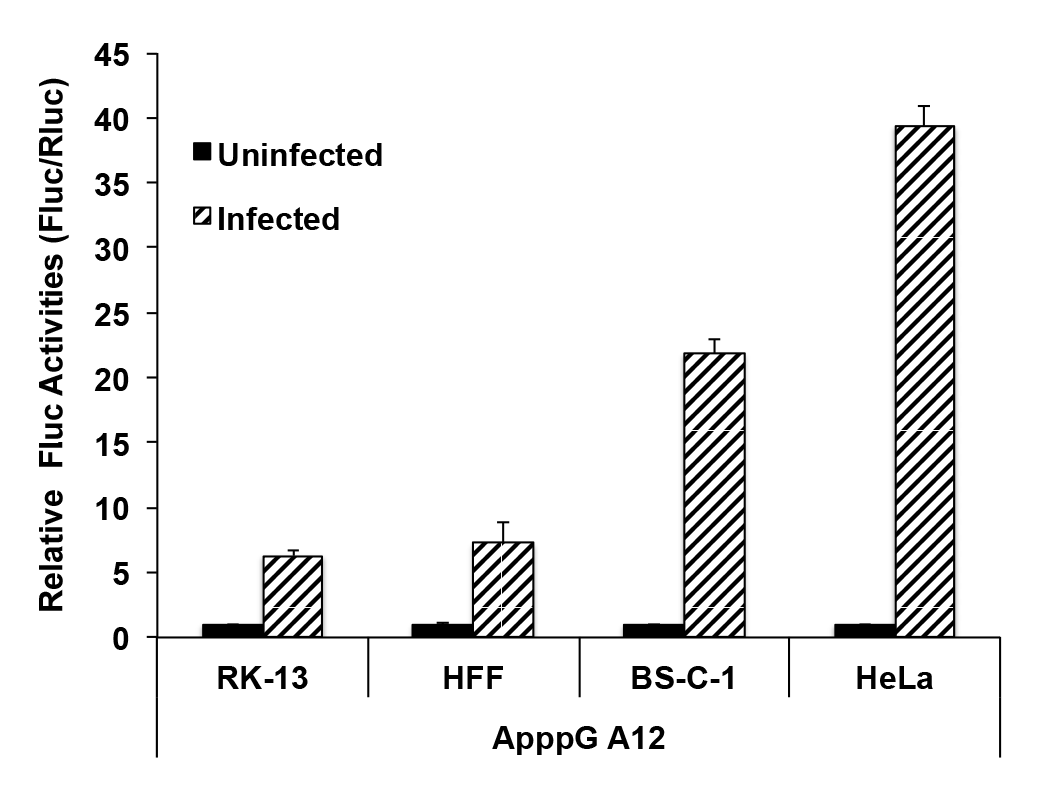

Supplement: S5 Fig — ApppG-capped, 12A-headed Fluc reporter mRNA was transfected into indicated uninfected and VACV-infected cells together with an m7G-capped Rluc mRNA. Luciferase activities were measured at 5 h post transfection. The Rluc normalized Fluc activities were normalized as 1 in uninfected cells. Error bars represent standard deviation (SD) of at least three experiments. (TIF) [file ppat.1006602.s005.tif]

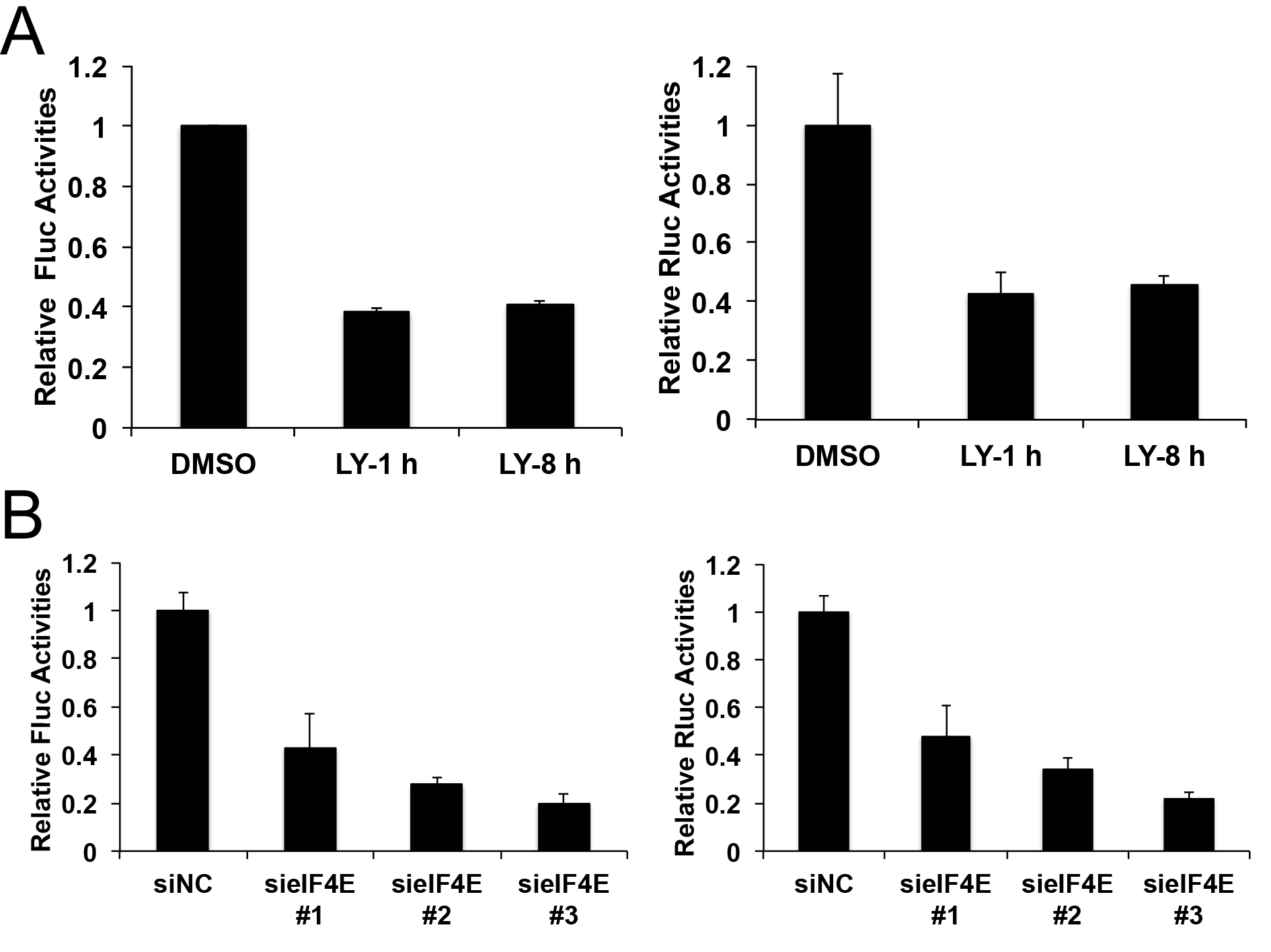

Supplement: S6 Fig — (A) HeLa cells were treated with DMSO or LY294002 at indicated times in mock-infected cells. An Fluc reporter mRNA headed with 12 As was transfected into uninfected cells together with an Rluc mRNA with a Kozak sequence-containing 5’-UTR at 12 hpi. Firefly (left) and renilla (right) luciferase activities were measured at 5 h post transfection. Luciferase activities were normalized as 1 in DMSO treated cells. (B) HeLa cells were transfected with control (siNC) or siRNAs targeting eIF4E for 48 h. An Fluc reporter mRNA headed with 12 As was transfected into uninfected cells together with an Rluc mRNA with a Kozak sequence-containing 5’-UTR at 12 hpi. Firefly (left) and renilla (right) luciferase activities were measured at 5 h post transfection. Error bars represent standard deviation (SD) of at least three experiments. Luciferase activities were normalized as 1 in siNC-transfected cells. Error bars represent standard deviation (SD) of at least three experiments. (TIF) [file ppat.1006602.s006.tif]
